# Yeast culture promotes butyrate produced fibrolytic bacteria as intracellular hydrogen sink in the rumen

**DOI:** 10.1186/s40168-026-02436-3

**Published:** 2026-05-25

**Authors:** Li Wang, Fei Li, Zhiyuan Ma, Emilio M. Ungerfeld, Tianxi Zhang, Zhian Zhang, Xiaozhen Liu, Qian Zhang, Xiumin Zhang

**Affiliations:** 1 State Key Laboratory of Herbage Improvement and Grassland Agro-Ecosystems, College of Pastoral Agriculture Science and Technology, Lanzhou University, Lanzhou, 730020 China; 2 Centro Regional de Investigación Carillanca, Instituto de Investigaciones Agropecuarias INIA, 4880000 Vilcún, Chile; 3 Institute of Grassland Research, Chinese Academy of Agriculture Sciences, Huhhot, 010010 China; 4 State Key Laboratory of Forage Breeding-By-Design and Utilization, Key Laboratory for Agro-Ecological Processes in Subtropical Region, Institute of Subtropical Agriculture, Chinese Academy of Sciences, Changsha, 410125 China

**Keywords:** Yeast culture, Butyrogenesis, Rumen, Hydrogen, Subacute ruminal acidosis

## Abstract

**Background:**

Yeast culture (YC) supplementation is widely adopted to mitigate rumen pH depression and alleviate the inhibition of fiber degradation under starch-rich diets. Yet, the underlying microbial mechanisms, particularly how yeast culture orchestrates fibrolytic communities and affects metabolic hydrogen flow in the rumen, remain a critical knowledge gap. Accordingly, elucidating the microbial basis by which yeast culture modulates fiber degradation and hydrogen utilization under starch-rich diets is of both theoretical and practical importance.

**Methods:**

We conducted a study with growing lambs receiving starch-rich diets that differed only in yeast culture supplementation (CON 0%, YC 1%). We evaluated their growth performance, apparent total-tract digestibilities, rumen fermentation end-products, and the rumen metagenome.

**Results:**

The YC treatment increased the lambs’ final body mass (*P* = 0.02), average daily gain (*P* = 0.03), digestibilities of neutral detergent fiber (*P* < 0.001) and acid detergent fiber (*P* < 0.001), and rumen pH (*P* < 0.05), and tended to increase organic matter digestibility (*P* = 0.09). In addition, total VFA concentrations, particularly butyrate, were higher at 6 h post-morning feeding (*P* = 0.01). Fibrolytic and hydrogenotrophic taxa (e.g., *Ruminococcus*_E and *Quinella*) and CAZyme families, including GH43, GH31, GH9, and GH35, were enriched by the YC treatment, as were bacteria involved in fiber degradation and butyrate production. Furthermore, none of the top five YC treatment-enriched bacterial genomes contained any hydrogenase genes, which indicates that this butyrogenic fibrolytic consortium is significantly different from the hydrogen-producing fiber-degrading microorganisms we are familiar with.

**Conclusion:**

Yeast culture supplementation promoted the proliferation of a distinct butyrogenic consortium that degrades fiber while apparently disposing intracellularly metabolic hydrogen generated during fermentation, rather than releasing it as H_2_. These findings provide a microbial basis for understanding how yeast culture improves fermentation efficiency under starch-rich diets and suggest that selecting yeast culture products capable of promoting butyrogenic fibrolytic bacteria may be beneficial for ruminant performance and rumen stability.

Video Abstract

**Graphical Abstract:**

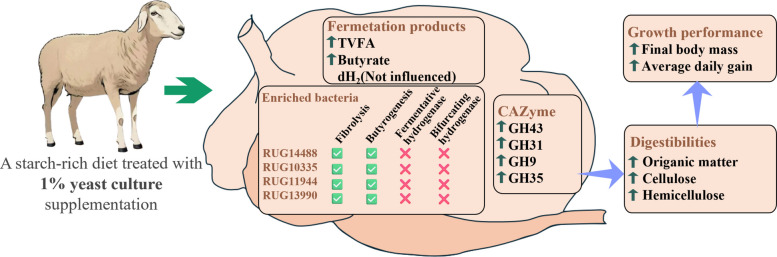

**Supplementary Information:**

The online version contains supplementary material available at 10.1186/s40168-026-02436-3.

## Background

Increasing starch content in diets is a common practice in modern livestock production to meet the growing demand for meat and dairy products. High starch levels rapidly supply animals with the energy needed for growth. However, this approach often leads to excessive accumulation of volatile fatty acids (VFA) and lactic acid in the rumen, thus increasing the risk of subacute ruminal acidosis [1–3]. Meanwhile, pelleted total mixed rations are increasingly used in ruminant production due to benefits such as ease of transportation, storage stability, and avoidance of feed fractions selection [4]. In pelleting, coarse forage is ground to particles smaller than 8 mm, which reduces the physically effective fiber content of the diet and consequently decreases rumination and chewing times [5]. Both practices are widespread in sheep farming and directly contribute to lower rumen pH, the onset of ruminal acidosis, and reduced fiber degradation.

Studies have demonstrated that supplementing ruminants fed starch-rich diets feed with yeast culture products rich in bioactive compounds, including oligosaccharides, amino acids, peptides, proteins, organic acids, vitamins, minerals, and enzymes [6], enhances ruminants’ production performance and improves rumen pH homeostasis along with the activities of key fibrolytic enzymes such as avicelase, carboxymethyl cellulase (CMCase), cellobiase, and xylanase [7–9]. Yeast culture products play a crucial role in reshaping the rumen microbial community, notably through increasing the abundance of fibrolytic bacteria [10]. Long-term in vitro fermentation study showed that yeast culture supplementation enhanced maize straw degradation and VFA production [11]. Although the relative abundance of *Ruminococcus* decreased, yeast culture promoted the growth of other microbial populations, notably *Prevotella*, *Succinicasticum*, *Treponema*, *Olsenella*, *Pyramidobacter*, and *Oribacterium*, among which *Prevotella* plays a key role in hydrolyzing xylan, xyloglucan, and pectin and converting these substrates into acetate, succinate, and propionate. However, our current understanding of these microbial processes is largely limited to taxonomic shifts identified via 16S rRNA gene amplicon sequencing [3]. To date, studies examining the impact of yeast cultures on the rumen metagenome that provide insights on functional potential and metabolic pathways remodeling are lacking.

The disposal of metabolic hydrogen ([H]) in the rumen is a crucial process that ensures the re-oxidation of redox coenzymes, making it an essential requisite for efficient rumen fermentation [12, 13]. During the anaerobic fermentation of rumen microorganisms, [H] in reduced cofactors can be transferred intracellularly to fermentation intermediates [14]. Alternatively, [H] can produce H_2_ and then be transferred to H_2_-consuming microorganisms through interspecies H_2_ transfer [15]. Metagenomic analysis has confirmed that the predominant pathways for microbial [H] disposal differ depending on the type of dietary carbohydrate, with [H] disposal through interspecies H_2_ transfer decreasing with high-starch diets [16]. Interestingly, dietary yeast culture supplementation under starch-rich conditions has resulted in elevated molar proportions of propionate [3, 17] or butyrate [7, 8], both being pathways effectively consuming [H] in rumen fermentation. However, the mechanism through which yeast culture affects the flow of [H] in the rumen under high starch conditions remains unclear.

While it is empirically established that yeast culture enhances rumen fermentation under high-starch conditions, the microbial mechanisms driving its benefits—especially its dual effect on redirecting [H] flow—remain poorly characterized. We hypothesized that yeast culture selectively enriches a fibrolytic consortium and shifts the partitioning of [H] toward propionate and/or butyrate formation. To test this, *Hu* lambs were fed starch-rich diets with (YC 1%) or without (CON 0%) yeast culture supplementation, followed by a systematic evaluation of growth performance, nutrient digestibility, and rumen fermentation parameters integrated with rumen metagenomic profiles.

## Results

### The YC treatment improves growth performance and fiber digestibility

Final body mass and average daily gain (ADG) were increased (*P* ≤ 0.02, Table 1) by YC treatment compared with CON, starting under an equivalent initial body weight condition. Dry matter intake (DMI) was not affected by treatment (*P* = 0.61). Feed efficiency expressed as DMI/ADG was decreased (*P* = 0.02) by YC treatment compared with CON. Moreover, the digestibilities of neutral detergent fiber (NDF) and acid detergent fiber (ADF) were increased by YC treatment compared to CON (*P* < 0.001), and organic matter (OM) digestibility tended to increase (*P* = 0.09). In addition, the apparent digestibility of hemicellulose increased from 45.9% (CON) to 51.7% (YC) (*P* < 0.001), and cellulose increased from 26.3% to 41.5% (*P* = 0.04). Crude protein (CP) and ether extract (EE) digestibilities did not differ significantly between treatments (*P* ≥ 0.32).

**Table 1:** Effects of yeast culture supplementation on growth performance and apparent digestibility of growing lambs (*n* = 10)

Item	CON	YC	SEM	*P*-value
Growth performance				
Final BM, kg	37.5	39.6	0.57	0.02
ADG, g/d	225	271	10.6	0.01
DMI, kg/d	1.52	1.45	0.05	0.61
DMI/ADG, kg/kg	6.43	5.39	0.25	0.02
Apparent digestibility (%)				
OM	71.2	72.7	0.57	0.09
CP	75.4	76.5	0.81	0.32
NDF	37.6	45.6	0.46	< 0.001
ADF	27.4	36.4	0.61	< 0.001
Hemicellulose	45.9	51.7	0.84	< 0.001
Cellulose	26.3	41.5	4.78	0.04
EE	81.0	80.7	1.24	0.90
Energy	68.1	69.4	0.63	0.15

*CON* Control, *YC* 1% yeast culture supplementation, *BM* Body mass, *ADG* Average daily gain, *DMI* Dry matter intake, *DM* Dry matter, *OM* Organic matter, *CP* Crude protein, *NDF* Neutral detergent fiber, *ADF* Acid detergent fiber, *Hemicellulose* NDF – ADF, *Cellulose* ADF – ADL, *EE* Ether extract

### The YC treatment increases rumen VFA concentration

Rumen pH was higher in YC treatment than in CON (*P* < 0.01, Table 2), with no treatment × time interaction effect detected (*P* = 0.75). A treatment × time interaction was observed for total VFA and butyrate concentration (*P* ≤ 0.04), with both total VFA and butyrate being higher in the YC treatment than in CON at 6 h post-morning feeding (*P* ≤ 0.02). Concentrations of acetate, propionate, and acetate/propionate did not differ significantly between treatments (*P* ≥ 0.21). No significant treatment × time interaction (*P* ≥ 0.43) or treatment effect (*P* ≥ 0.22) was detected for dissolved H_2_ or dissolved CH_4_. Rumen ammonium nitrogen (NH_4_^+^–N) concentrations ranged from 7.59 to 19.2 m*M* across sampling time points and did not differ between treatments (*P* = 0.81; Table 2). Rumen lactate concentrations were low throughout the post-feeding period and were not affected by YC treatment (*P* = 0.65, Table 2).

**Table 2:** Effects of yeast culture supplementation on rumen fermentation in lambs (*n* = 10)

Item	0 h		2.5 h		6 h		SEM	*P*-value		
	CON	YC	CON	YC	CON	YC		Treatment	Time^2^	Treatment × Time
pH	6.56	6.87	5.63	5.95	5.88	6.09	0.10	< 0.01	< 0.01	0.75
VFA concentration, m*M*										
Total VFA	29.9	23.7	99.7	101	95.4^b^	118^a^	3.39	0.36	< 0.01	0.04
Acetate	17.7	15.7	51.5	53.2	44.5	55.8	1.42	0.21	< 0.01	0.06
Propionate	7.85	4.31	34.3	32.5	36.7	41.0	2.01	0.89	< 0.01	0.19
Butyrate	2.73	2.29	11.2	12.9	11.2^b^	17.2^a^	0.61	0.07	< 0.01	0.01
Others^1^	1.63	1.41	2.67	2.75	2.98	4.18	0.541	0.30	< 0.01	0.15
Acetate/propionate	2.26	3.64	1.50	1.63	1.21	1.36	0.371	0.22	< 0.01	0.11
NH_4_^+^, m*M*	7.65	7.59	19.2	18.9	11.1	12.1	1.448	0.81	< 0.01	0.79
Lactate, m*M*	0.17	0.21	0.63	0.54	0.36	0.37	0.052	0.65	< 0.01	0.13
Dissolved gases										
H_2_, μ*M*	0.38	0.03	1.85	0.85	7.10	5.14	1.559	0.22	< 0.01	0.77
CH_4_, μ*M*	100	124	21.4	17.2	58.6	28.7	28.27	0.82	< 0.01	0.43

^1^ Others include isobutyrate, isovalerate, 2-methylbutyrate, and valerate, with isovalerate and 2-methylbutyrate co-eluting the GC column

^2^ Rumen content samples were collected at three time points relative to feeding: pre-feeding (0 h), 2.5 h post-feeding, and 6 h post-feeding. ^ab^ Letter-based significance notation compares within sampling time points when treatment × time interactions were present (*P* < 0.05). Unlike superscripts within a time point indicate significant effects between treatments (*P* < 0.05)

### The YC alters the composition of the rumen microbiomes

The YC treatment had no significant effects on microbial alpha diversity indices across species (*P* ≥ 0.29, Fig. S1.A) and phyla (*P* ≥ 0.29, Fig. S2), but altered beta diversity at the species taxonomic rank (*R*^2^ = 0.29, *P* < 0.01; Fig. S1.B) with clear separation between CON and YC samples in the principal coordinates analysis (PCoA) plot of the top two coordinates of PCo1 and PCo2 explaining 34.4% and 9.6% of the variance, respectively. In the PCoA based on Bray–Curtis distances, samples from the YC treatment displayed greater within-group dispersion, whereas CON samples clustered more tightly. The taxonomic profile of rumen microbiomes from species to phylum rank is presented in Dataset S1. Species rank changes were concentrated within *Prevotella*, which accounted for 13 of the top 20 species by relative abundance. Among 13 *Prevotella* species, 9 differed between treatments: YC treatment increased three species, including *P.* sp002354095, *P.* sp900315525, and *P*. sp900318795 (*P* < 0.01; Dataset S1), and decreased six species, including *P. mizrahii* (*P* < 0.001) and *P.* sp900316565 (*P* < 0.01). In addition, within the top 20 species, YC treatment increased the relative abundance of *Quinella* sp 947165295 (*P* < 0.001).

### The YC treatment enriches genes involved in fiber degradation and butyrogenesis in rumen

**Fig. 1:**
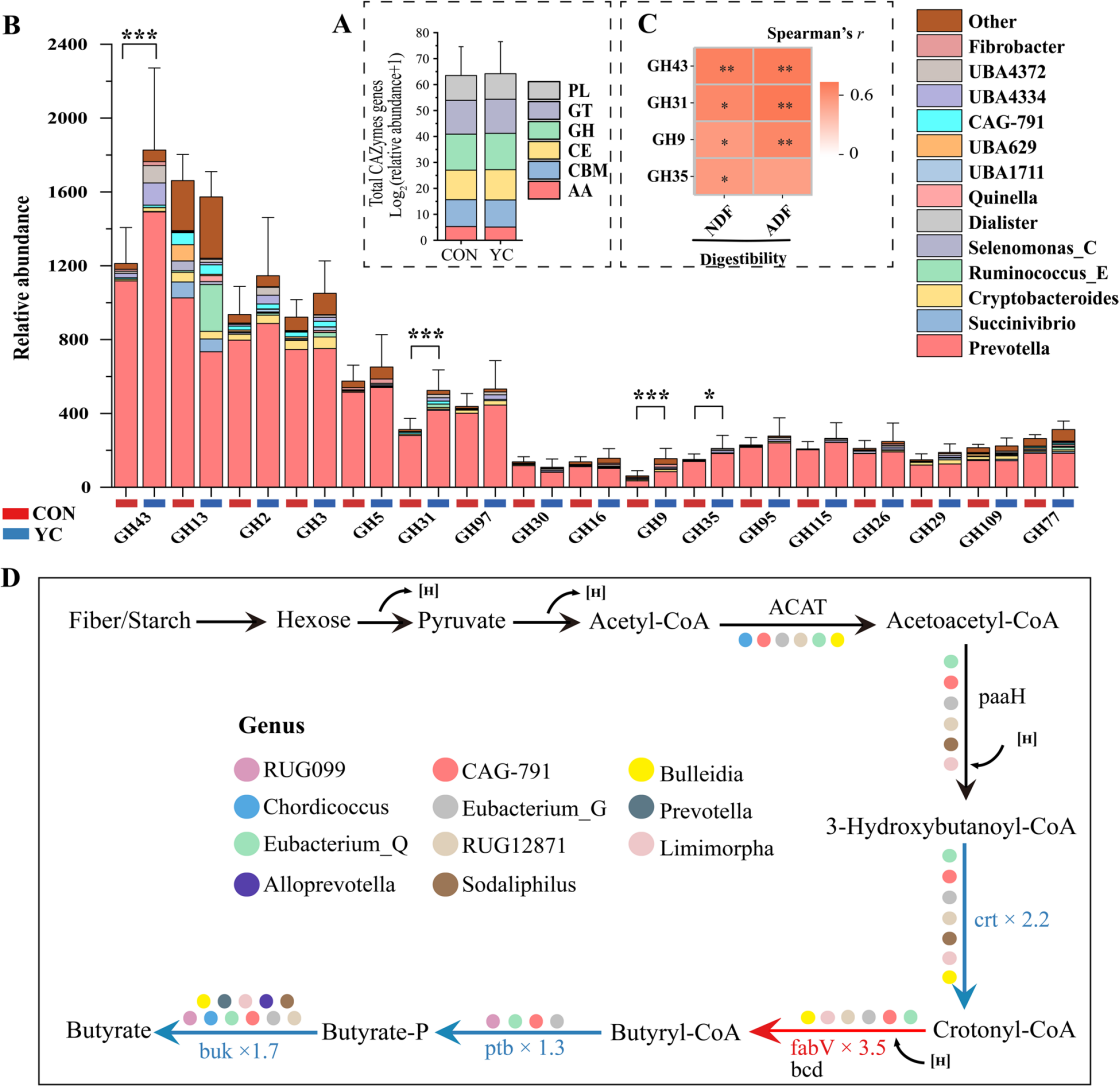
Effects of yeast culture supplementation on rumen metagenomic profiles involved in fiber degradation and butyrogenesis (*n* = 10). **A** Relative abundance of total CAZyme genes. **B** GHs gene families involved in cellulose and hemicellulose degradation; bar plot fill colors represent GH contributors at the genus rank (Dataset S2). **C** Correlations between affected GH families and fiber digestibility. **D** Butyrogenesis pathway and its contributors at genus rank. “ × 1.7,” “ × 1.3,” “ × 3.5,” and “ × 2.2” indicate fold change between treatments; blue denotes enrichment in YC and red denotes enrichment in CON

The results of all six CAZyme families, including glycoside hydrolases (GHs), glycosyl transferases (GTs), polysaccharide lyases (PLs), carbohydrate esterases (CEs), auxiliary activities (AAs), and carbohydrate-binding modules (CBMs), are presented in Dataset S2. The YC treatment exhibited no significant effects on the relative abundances of the six CAZyme families (*P* > 0.05, Fig. 1A), but increased specific GH subfamilies (Fig. 1B), including GH43 (1.4-fold, *P* < 0.001), GH31 (1.6-fold, *P* < 0.001), GH9 (2.5-fold, *P* < 0.001), and GH35 (1.3-fold, *P* = 0.02). These increases were mainly driven by *Prevotella*, *Succinivibrio*, *Ruminococcus*_E, CAG-791, UBA4334, UBA4372, and *Fibrobacter*. Four GH subfamilies significantly enriched by the YC treatment showed strong positive correlations with the digestibility of NDF (*r* ≥ 0.56, *P* ≤ 0.04; Fig. 1C).

The KEGG orthology (KO) profiles are presented in Dataset S3. A total of 35 pathways at level 2 were affected by the YC treatment. Carbohydrate metabolism was not altered (*P* = 0.63). At the level 3 classification, the YC treatment enriched the pyruvate metabolism pathway (*P* = 0.04).

The comprehensive results of genes encoding enzymes involved in major VFA production are presented in Dataset S4. Among all eight genes investigated for acetate production, phosphate acetyltransferase (*pta*) was downregulated by YC treatment (*P* = 0.01, Fig. S3). This decrease was consistent with a reduced contribution of UBA629 to the *pta* signal in the YC treatment, suggesting that the lower *pta* abundance may partly reflect a diminished contribution from this genus to the pathway of acetate formation. None of the genes involved in propionate’s acrylate (non-randomizing) pathway was affected by the YC treatment (*P* ≥ 0.75, Fig. S4). However, in propionate’s succinate (randomizing) pathway, seven genes were enriched (*P* ≤ 0.05) while four genes were depleted (*P* ≤ 0.05) by the YC treatment. The observed enrichment of *oadB*, *maeA*, *fumA*, and *fumB* was consistent with increased contributions originating from the taxa *Ruminococcus*_E and *Quinella* to these respective gene signals. Among the various key butyrogenesis genes (Fig. 1D), the relative abundances of enoyl-CoA hydratase (*crt*; *P* = 0.001, 2.2-fold increase), phosphate butyryltransferase (*ptb*; *P* < 0.001, 1.3-fold), and butyrate kinase (*buk*; *P* < 0.05, 1.7-fold) genes were enriched by the YC treatment. Specifically, the enrichment of *buk* in the YC treatment was primarily attributed to genera *Prevotella*, *Cryptobacteroides*, UBA1711, and CAG-791 (Fig. S5). We also screened marker genes of the Wood–Ljungdahl pathway: the relative abundance of *acsA* was lower in the YC treatment (*P* < 0.001), whereas *acsB* and other acetyl-CoA synthase subunits showed no differences (*P* > 0.05; Dataset S4).

**Fig. 2:**
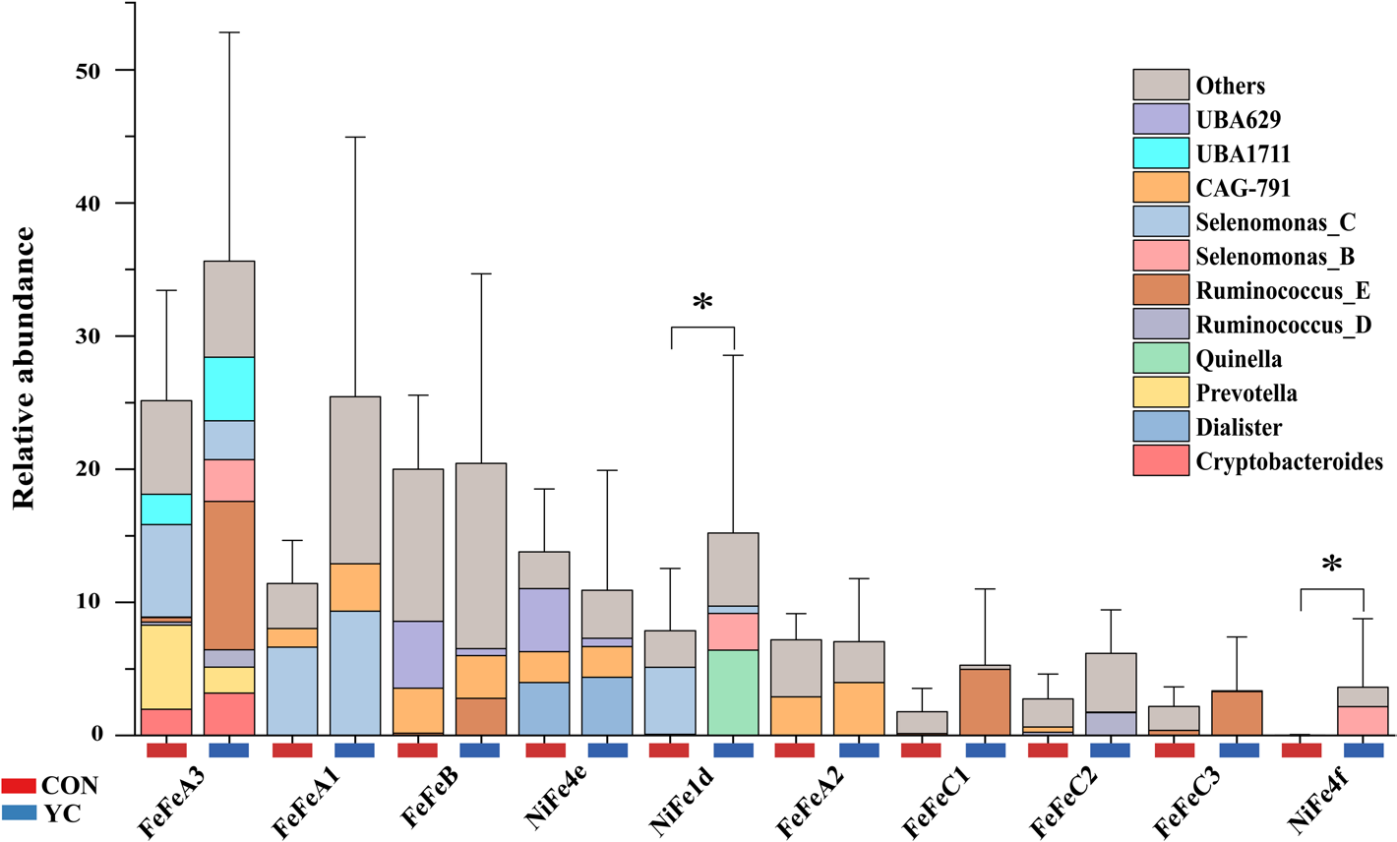
Hydrogenases and their contributors at the genus rank. Fermentative hydrogenases (group A1, A2 and B FeFe-hydrogenases), electron-bifurcating hydrogenases (group A3 FeFe-hydrogenases), energy-converting hydrogenases (bidirectional; group 4f NiFe-hydrogenases), respiratory hydrogenases (group 1 d NiFe-hydrogenases), sensory hydrogenases (group C FeFe-hydrogenases). *P < 0.05, *n* = 10/group

Results of hydrogenases-encoding genes are presented in Dataset S2. The [FeFe]-A3-bifurcating hydrogenase genes were the most abundant in both treatments and were unaffected by the YC treatment (*P* = 0.14, Fig. 2). The YC treatment enriched genes encoding [NiFe]−1d (*P* = 0.03) and [NiFe]−4f hydrogenases (*P* = 0.01).

### The microbiomes associated with butyrogenesis coincide with the microbiome associated with increased NDF and ADF digestibilities

To further investigate the effect of YC treatment at the species rank, we performed a prokaryote analysis (Fig. S6). We assembled 201 MAGs from our rumen microbiota samples and integrated them into the superset publicly available rumen microbiomes catalog [18].

After removing redundant entries at the species rank and applying quality control filters (completeness ≥ 80% and contamination ≤ 10%), a final set of 2750 high-quality rumen microbiomes was obtained. After Genome Taxonomy Database (GTDB) annotation (Dataset S5), a total of 26 phyla were represented, with Bacillota_A (1196 MAGs), Bacteroidota (733 MAGs), and Bacillota_I (211 MAGs) being the most dominant (Fig. S6). Additionally, 51 MAGs were archaeal, classified into Methanobacteriota and Halobacteriota (Dataset S5).

**Fig. 3:**
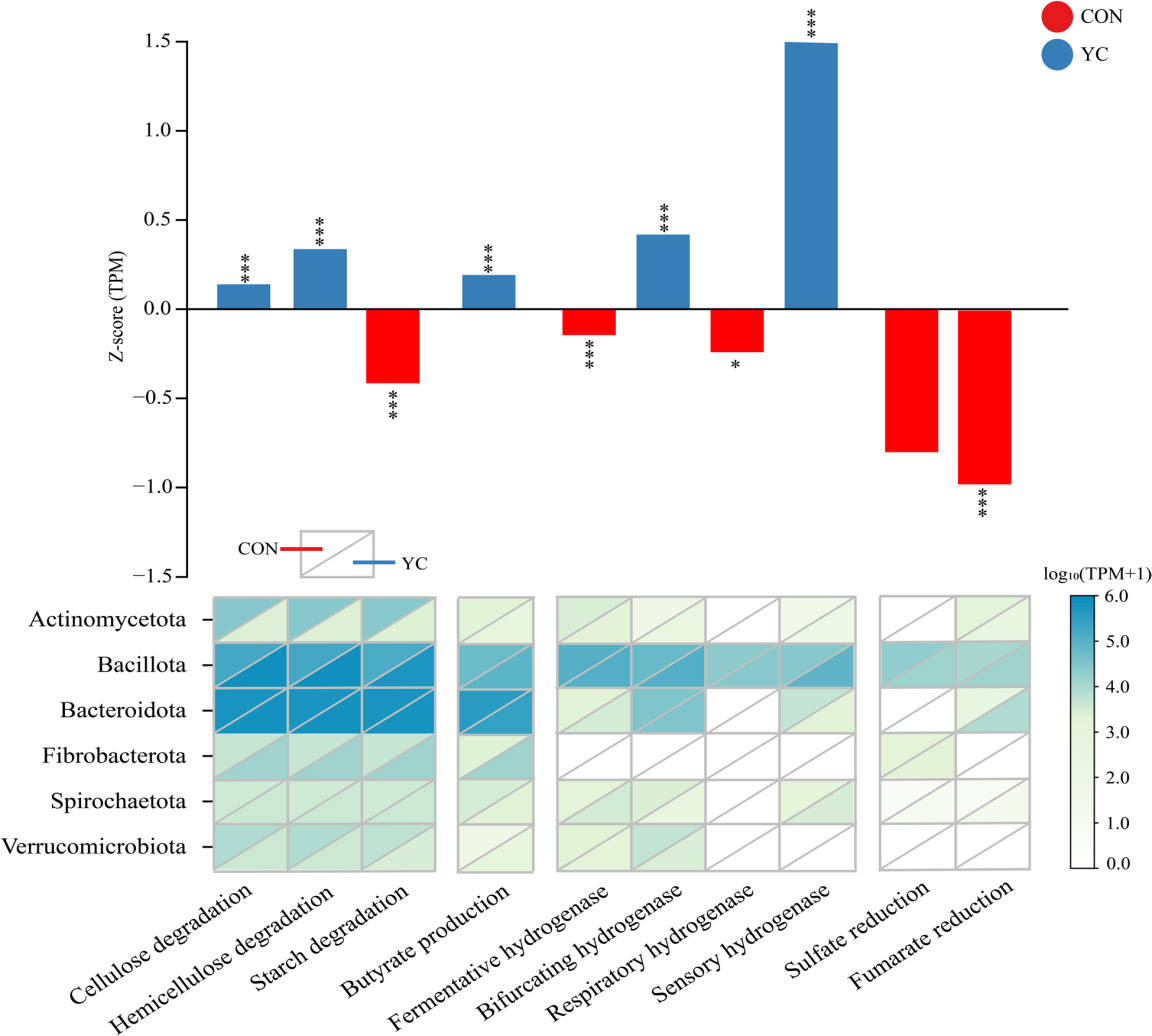
Functional stratification of microbial MAGs enriched by CON and YC treatment. Functional divergence of fibrolytic and butyrogenic microbial functional groups between CON and YC treatments (*n* = 10). Heatmap depicts *Z*-scores-normalized abundance of key microbial phyla and their associated enzymatic profiles (log_10_[TPM + 1] transformed) involved in fiber degradation and butyrogenesis. * *P* < 0.05, ** *P* < 0.01, *** *P* < 0.001

We functionally annotated the MAGs and assessed their potential in carbohydrate degradation, butyrate production, and H_2_ metabolism. The results of gene distribution in microbiomes are presented in Supplementary Dataset S5. Most bacterial MAGs encoded GH enzymes for carbohydrate degradation (Fig. S6). Specifically, 86.5% encoded enzymes for cellulose degradation, 81.9% for hemicellulose degradation, and 85.3% for starch hydrolysis. The YC treatment enriched the GH9, GH31, GH35, and GH43 families (*P* < 0.05). These GH families associated with cellulose and hemicellulose degradation were predominantly encoded by genomes assigned to Clostridia, Bacteroidia, and Bacilli. The YC treatment also increased the abundance of butyrate producers, including MAG5 and MAG148 (*P* < 0.05). Among 424 MAGs encoding *buk*, 111 MAGs were enriched by YC treatment (*P* < 0.05), most of which belonged to Bacillota and Bacteroidota (Fig. 3; Dataset S5). Approximately 63% of bacteria had the genetic capacity for H_2_ metabolism, including various hydrogenase subgroups and terminal reductases. The YC treatment enriched bifurcating and sensory hydrogenases (Fig. 3, *P* < 0.001). Notably, the YC treatment enriched Bacillota genes encoding sensory hydrogenases ([FeFe]-C hydrogenases), and Bacillota and Bacteroidota encoding genes for cellulose and hemicellulose degradation, butyrate production, and bifurcating hydrogenases.

**Fig. 4:**
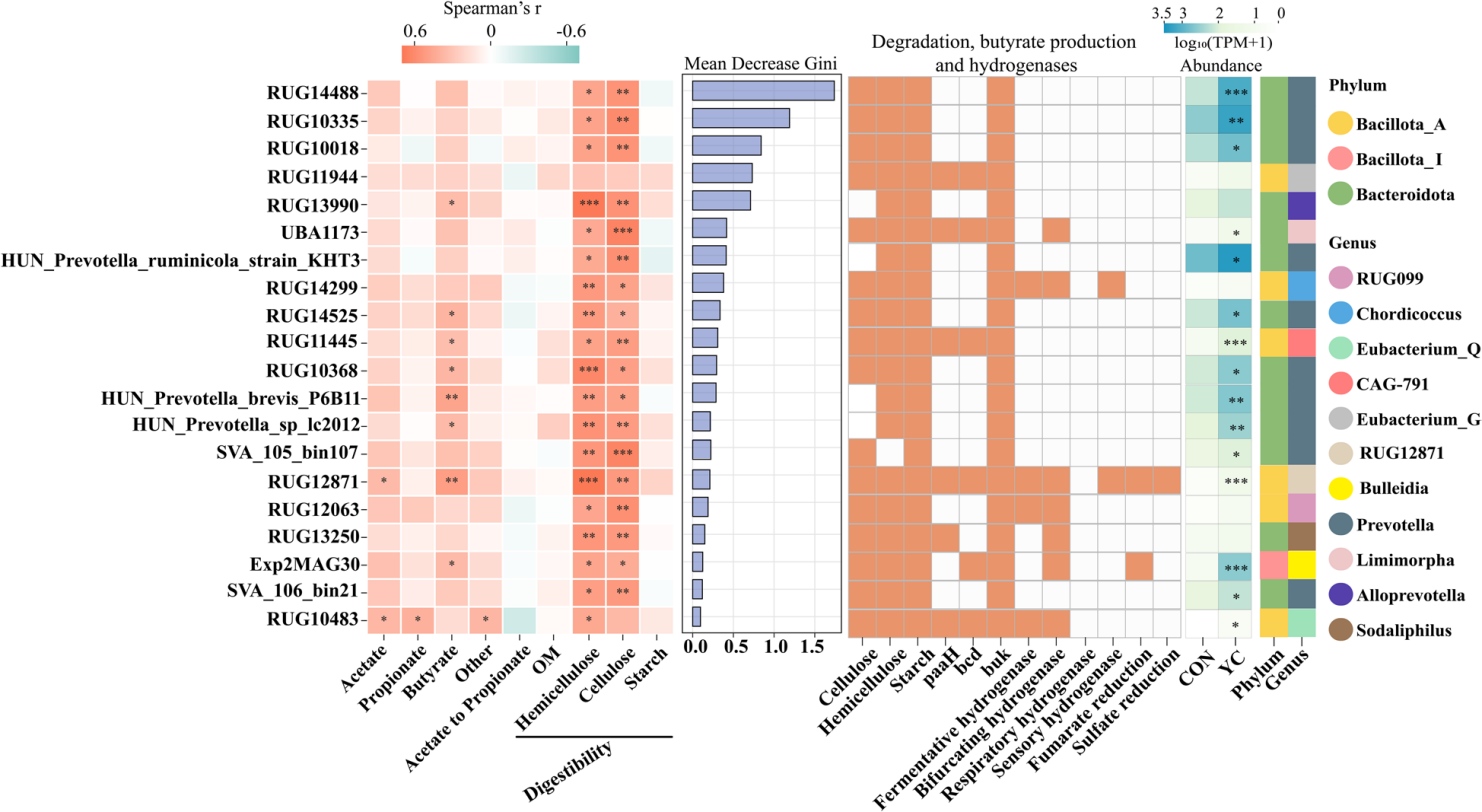
Heatmaps illustrating YC-enriched microbial taxa. Left panel: Spearman correlations between YC-enriched microbial taxa and two metabolic outputs: Volatile fatty acid concentrations (m*M*) and nutrient digestibility. Middle panel: Machine learning-based prioritization of MAGs. The accompanying bar plot ranks microbiomes based on their importance scores (Mean Decrease Gini index) from the random forest analysis (500 trees, OOB error = 0.47). Right panel: Landscape of functional genes involved in nutrient digestibility, butyrogenesis, and hydrogenesis in YC-enriched MAGs

We identified the top 20 MAGs positively correlated to rumen butyrate concentration and NDF and ADF digestibilities, and then ranked them by the Mean Decrease Gini index in the random forest model (Fig. 4). Mean Decrease Gini is a commonly used measure of feature importance in random forest models; taxa with higher values contribute more to prediction and are therefore ranked as more important. Among the top 20 MAGs in the yeast treatment, 13 contained no hydrogenase genes, and the five MAGs with the highest Gini index rankings lack any hydrogenase genes. Seventeen MAGs were positively correlated with NDF and ADF digestibilities (e.g., RUG14488; *r* > 0.55, *P* < 0.05). Eight MAGs were positively correlated with butyrate concentration (e.g., Exp2MAG30; *r* > 0.48, *P* < 0.05). More importantly, all eight MAGs positively correlated with butyrate concentration were also positively correlated with NDF and ADF digestibilities (*r* > 0.53, *P* < 0.05). At the phylum rank, these MAGs were predominantly assigned to Bacillota_A, Bacillota_I, and Bacteroidota (Fig. 4). At the genus rank, they were mainly affiliated with *Prevotella*, CAG-791, and *Bulleidia*. The three top-ranked MAGs (RUG14488, RUG10335, and RUG10018) were more abundant in the YC treatment (*P* < 0.05) and exhibited strong positive correlations with NDF and ADF digestibilities (*r* > 0.56).

## Discussion

The present research confirmed that yeast contributed to stabilization of rumen pH which stimulated fibrolytic bacteria and improved fiber digestion. With starch-rich diets the rumen is prone to prolonged low-pH states, which decrease fiber degradation [19], as fibrolytic bacteria are sensitive to low pH [20–22]. In agreement, Qi and Wang [6], Dias et al. [23] and Knollinger et al. [24] reported that yeast culture supplementation can enhance feed intake and growth performance under starch-rich diets, which is attributed to stabilization of rumen pH [3]. We hypothesized that under a high starch diet, through pH stabilization, yeast culture would stimulate fibrolytic bacteria producing propionate and/or butyrate as sinks of [H] instead of H_2_. Our earlier research using continuous rumen pH monitoring and in situ forage degradation assays had confirmed that yeast culture contributes to stabilizing rumen pH [8]. It is acknowledged that in comparison, oral tubing, as conducted in the present study, overestimates rumen pH because of inevitable saliva contamination. Yet, as the daily pattern of evolution of rumen pH obtained using oral tubing does not seem to be altered in comparison with sampling via rumen cannulae [25], we consider that, even if absolute pH is likely overestimated because of the sampling method, yeast supplementation still ameliorated the low rumen pH prevalent with the high starch diet.

Although yeast culture is often used in starch-rich diets to mitigate ruminal acidosis and the concomitant depression in fiber degradation, mechanistic research suggests that its effects may involve more than pH stabilization alone. Yeast-based products have been proposed to scavenge oxygen associated with newly ingested feed [26], and to stimulate lactate uptake through the provision of dicarboxylic acids [27, 28]. In the present study, however, lactate concentrations were small and unaltered by YC treatment. The effects of yeast culture on oxygen sequestration cannot be evaluated in the present work as a possible mechanism of stimulation of fibrolytic bacteria, as neither dissolved oxygen concentration nor redox potential was determined. Dietary starch can also depress NDF degradation through other pH-independent mechanisms such as through stimulating microbial populations that compete with fibrolytic microorganisms for branched-chain VFA, and through impaired adhesion of cellulolytic microorganisms after feeding [29]. Whether or not a relief of those mechanisms might have played a role on the positive response of fiber digestion to yeast culture supplementation in this study and others is unclear.

It has been empirically observed that yeast culture supplementation has increased propionate [3, 17] and/or butyrate [7, 8, 30, 31] concentrations in the rumen. Yeast culture contains dicarboxylic acids such as malate and fumarate, which function as electron acceptors if metabolized to propionate, providing a mechanistic basis to explain increased propionate concentration [32]. However, meta-analyses of results obtained in in vitro cultures concluded that exogenously added fumarate [33] and malate [34] are converted to propionate with low efficiency, and that the incorporation of [H] was considerably lower than theoretical due to simultaneous release of [H], as part of the fumarate and malate seemed to be converted to acetate. Furthermore, the small amount of malate reported to be present in yeast (1.73 to 2.62% DM; [35]), along with the small inclusion of yeast culture in ruminant diets, makes doubtful the direct contribution of intermediates of propionate production present in YC to the observed increases in propionate concentration obtained with yeast culture supplementation in some studies.

Our findings indicate that pH stabilization stimulated fibrolytic bacteria that utilize butyrate as their main electron sink. The mechanism through which yeast culture stimulates butyrate producers, resulting in increased butyrate concentration, is also unclear. A possible explanation for the differing results among studies in terms of which fermentation pathway is stimulated by yeast lies in their content of B-vitamins [36], as supplementation with yeast can increase B-vitamin concentration in the rumen [37]. Both biotin and cyanocobalamin are required in the randomizing pathway of propionate formation [38–40]. In turn, riboflavin is a precursor of flavins [41], which are involved in flavin-based electron bifurcation in crotonyl-CoA reduction to butyryl-CoA in butyrate formation [42]. We speculate that whether supplemental yeast predominantly stimulates propionate or butyrate production in different studies may partly depend on balance between the supply of B-vitamins present in different yeast cultures and the rumen availability of different B-vitamins with each basal diet, which may or may not impose limitations for each fermentation pathway. This said, at present, a mechanistic explanation for the variable fermentation responses to yeast supplementation is still not demonstrated. Future studies combining chemical characterization of yeast cultures with targeted in vitro or in vivo fermentations are required to identify the specific bioactive components driving the distinct fermentation responses.

In terms of functional composition, our metagenomic results were consistent with the elevated rumen butyrate concentration. The YC treatment enriched canonical butyrogenesis genes (*crt*, *ptb*, and *buk*) (Fig. 1D; Fig. S5), supporting a primary role for bacterial butyrate-forming pathways in the observed response. Nevertheless, we acknowledge a possible alternative explanation involving rumen ciliate protozoa, which also contribute to butyrate production [43]. While unquantified due to the limitations of ciliate metagenomics, protozoa represent plausible contributors to the observed butyrate increase, although the specific influence of yeast culture supplementation on their abundance or activity in this study remains unclear.

Among the 168 pure-cultured rumen butyrate-producing bacteria in the Hungate1000 project, only 4 strains have cellulose-decomposing ability, while 97 strains have the ability to decompose xylan [44]. Unlike this result, our study found that among the 20 most predominant microbial genomes enriched by the YC treatment, almost all had the ability to decompose both cellulose and hemicellulose. It is possible that fiber degradation and butyrogenesis were carried out by the same microorganisms.

Interspecies H_2_ transfer is an important mechanism for fiber degradation in the rumen, as the catabolism of monomers resulting from fiber degradation produces predominantly acetate, with excess [H] being converted into H_2_ via hydrogenases [12]. Dihydrogen so generated can be consumed by multiple hydrogenotrophic pathways in the rumen, including methanogenesis, reductive acetogenesis and fumarate reduction [15, 45]. Butyrate producers can also utilize H_2_ generated by other microbes, requiring hydrogenases to convert it into reduced redox cofactors [12]. However, we observed that neither the composition of major hydrogenases nor dissolved H_2_ concentrations were affected by YC treatment. When the competition for H_2_ by pathways alternative to methanogenesis increases, we would expect methanogenesis to decrease; however, our results do not support that expectation, as YC treatment did not affect methanogens and the methanogenesis marker gene *mcr*A. The underlying reason for this result is at present unclear and left open for further investigation. Also, marker genes for the Wood–Ljungdahl pathway showed no significant differences between treatments, suggesting that YC treatment did not enhance reductive acetogenesis. Furthermore, none of the top five YC treatment-enriched bacterial MAGs contained any hydrogenase genes. These results align with the hypothesis that both fiber degradation and butyrate production could have occurred in the same cell. This interpretation is supported by our genome-resolved analysis (Fig. 4; Dataset S5), where the candidate MAGs most closely associated with both butyrate concentration and apparent NDF and ADF digestibilities were mainly affiliated with *Prevotella*, CAG-791 and RUG12871. These candidates encoded both plant polysaccharide-degrading CAZyme repertoires and key butyrate-formation genes while lacking detectable hydrogenase genes, consistent with redox balancing taking place within cells rather than through an increase in interspecies H₂ transfer.

pH stabilization may influence not only fibrolytic activity but also the way rumen communities assemble across animals, which is reflected in the PCoA patterns. The PCoA plot showed greater within-group dispersion in the YC samples than in the CON samples. A plausible explanation is that the high-starch diet imposes a stronger low-pH stress in CON, which acts as a selective constraint and promotes a more convergent community configuration across individuals. This aligns with findings in human gut ecosystems, where significant dietary changes serve as environmental filters that drive microbial communities toward a more deterministic and convergent assembly [46]. By attenuating the pH challenge, YC may relax this constraint, allowing multiple alternative community states to persist while maintaining the overall fermentation.

In our metagenomic analysis, GHs, which catalyze the hydrolysis of glycosidic bonds, represented the most abundant CAZyme class at the gene rank, consistent with previous studies [20, 47]. Among the four GH families enriched by the YC treatment, GH43 decomposes the side chains of arabinan and xylan, GH31 is mainly involved in the hydrolysis and transglycosylation of α-glycosidic bonds, GH9 is mainly an endoglucanase, and GH35 mainly functions as a β-galactosidase [48]. Other studies reporting increases in fiber degradability following yeast culture supplementation [49] also observed an enrichment of GH43, one of the most abundant GH families in the rumen [30]. Because hemicellulose intertwines cellulose through highly branched side chains and also connects to pectins, enabling the three to form an ordered composite structure [50], hemicellulose degradation is an important step for rumen microorganisms to deconstruct the plant cell wall structure. GH43 enzymes are thus important for the initial stage of hemicellulose degradation, serving as mediators in the breakdown of the network of complex polysaccharides in the cell wall [51, 52]. Importantly, high-starch diets have been reported to shift CAZyme profiles away from fibrolytic functions, with GH43 being more abundant with fiber- than with starch-rich diets [16, 53]. Thus, we interpret the YC-associated GH43 increase as one explanation for how yeast culture supplementation improves fiber degradation with starch-rich diets.

Mewis et al. [54] identified 4555 GH43 sequences in the CAZy database, of which 4197 originated from bacteria and 312 from eukaryotes. Genomic analysis of rumen ciliates isolated from Holstein cattle, Qinchuan beef steers, and Guanzhong dairy goats revealed the presence of several GH43 (xylanase, EC 3.2.1.8) genes acquired through horizontal gene transfer [16]. However, these ciliate-derived GH43 proteins lack the CBM22 domain commonly found in bacterial GH43, which may decrease their cellulose-binding ability. In contrast, a meta-transcriptomic analysis of the bovine rumen found that nearly all detected GH43‑related transcripts originated from bacteria while anaerobic fungi and ciliates made no contribution to the expression of the genes encoding these enzymes [55]. However, rumen ciliates nonetheless play roles in fiber degradation via alternative enzymatic pathways. Rumen ciliates isolated from cattle and goats are enriched in various CAZymes—such as the cellulolytic enzymes GH5, GH9, and GH26 [56]. Ciliates can thus also contribute to plant cell-wall deconstruction by expressing a broad repertoire of fibrolytic CAZymes such as cellulase- and xylanase-related GHs detected in *Epidinium* proteomes [57]. Considering their fibrolytic role, a limitation of the present work is the lack of determination of ciliates’ abundance and activity.

Yeast culture enriched Bacillota-encoded CAZyme genes involved in cellulose and hemicellulose degradation (Fig. 3), consistent with observations in beef cattle fed diets with a higher forage-to-concentrate ratio [58]. Cellulose-associated CAZymes were mainly represented by GH9 along with multiple GH5 subfamilies, including GH5_38 and GH5_46, which were significantly increased with YC treatment. Because reference genomes for rumen eukaryotes—particularly anaerobic fungi—remain limited and eukaryotic-focused metagenomic binning/annotation tools (e.g., EukCC and EukRep) are still under development, the present analysis was restricted to bacteria, and potential eukaryotic contributions to cellulolytic CAZyme repertoires may not be fully captured.

In the present study, however, YC treatment did not measurably alter either rumen NH_4_^+^–N or lactate concentrations (Table 2), and *ldh* genes likewise showed no treatment effect (Dataset S4). Furthermore, it was observed that NH_4_^+^–N concentration remained above the minimum level typically considered necessary to sustain microbial growth, suggesting that rumen microorganisms were unlikely to be limited by nitrogen limitation [59, 60]. Our results are fully consistent with previous reports of ruminants adapted to high-starch diets [61, 62], lactate concentrations remained low, indicating no evidence of lactate accumulation. Even though we do not rule out the possibility of saliva contamination occurring as a consequence of rumen sampling using oral tubing [25, 63], low lactate concentrations suggest that pH might not have been extremely low even in the CON. It appears therefore, the very low acetate/propionate ratios observed in this work could be mediated partially but not entirely by low pH after feeding, and other factors, such as relatively high rumen outflow rates of concentrates [64–67], could have also contributed.

Compared with starch-rich diets, fiber-rich diets typically enrich fibrolytic bacteria and methanogens and are associated with a lower rumen dissolved H₂, consistent with methanogenesis acting as a major H₂ sink; on the other hand, rumens adapted to starch-rich diets have higher dissolved H₂ than those adapted to fiber-rich diets [16]. Li et al. [16] showed that a fiber-rich diet increased the abundance of genes encoding [FeFe]‑ and [NiFe]‑hydrogenases involved in H_2_ uptake for methanogenesis and reductive acetogenesis. Under these conditions, some butyrate-producing bacteria can convert acetate generated via the Wood–Ljungdahl pathway into butyrate [16, 53, 68]. However, this specific route was not supported as a YC-responsive mechanism in our results, as neither the Wood–Ljungdahl pathway marker *acsB* nor the CoA-transferase route gene *atoD* differed significantly between treatments; instead, YC-enriched *Eubacterium*_Q MAGs predominantly encoded *buk* while generally lacking *atoD*, suggesting a shift more consistent with *buk*-associated butyrate formation potential. Earlier studies suggested that live yeasts or yeast culture may stimulate reductive acetogenesis [69, 70]. In our study, however, screening of the Wood–Ljungdahl pathway markers did not support an increase of reductive acetogenesis by YC treatment as *acsB* was unchanged, and *acsA* decreased by the YC treatment. Although [NiFe]−1d hydrogenase genes mainly assigned to *Quinella* and *Selenomonas_B* were enriched by YC treatment, the abundance of genes encoding for other major hydrogenases genus such as [FeFe]-A3 showed no significant changes and dissolved H₂ concentrations remained unchanged. Taken together, these observations are consistent with the view that YC treatment may have promoted fiber degradation and shifted rumen fermentation toward butyrate, potentially through within-cell redox balancing, rather than being driven by a broad restructuring of interspecies H₂ transfer.

## Conclusion

This study used a metagenomic approach to analyze the microbial processes through which yeast culture can promote rumen bacterial fiber degradation and butyrogenesis with a starch-rich diet. Yeast culture supplementation promoted the proliferation of butyrogenic microorganisms degrading fiber while apparently intracellularly disposing of metabolic hydrogen generated during fermentation, rather than releasing it as H_2_. These findings provide a microbiological basis for understanding how yeast culture improves fermentation efficiency with starch-rich diets and suggest that selecting yeast culture products capable of promoting butyrogenic fibrolytic microorganisms may be beneficial for ruminant performance and rumen stability. Increased rumen butyrate is not only linked to improved growth performance but also serves as a primary energy source for rumen epithelial cells, promoting papillae development and absorptive capacity. The mechanisms through which yeast culture stimulated butyrate-producing bacteria remain to be elucidated.

## Materials and methods

### Experimental design and animal management

The study was approved by the Animal Care and Use Committee of Lanzhou University, Lanzhou, China (approval number CPAST-2023-MA-006). Under a completely randomized design, twenty male lambs (two‑month‑old, initial body mass: 22.62 ± 1.53 kg) were randomly allocated to two dietary treatments of starch-rich diets without (CON 0% DM) or with (YC 1% DM) yeast culture supplementation (Table S1). Both treatments received a starch-rich pelleted total mixed ration formulated with corn straw and a concentrate mixture. On a DM basis, the basal diet contained corn straw (200 g/kg), corn grain (250 g/kg), barley (250 g/kg), corn germ meal (105 g/kg), soybean meal (80 g/kg), cottonseed meal (50 g/kg), molasses (30 g/kg), limestone (15 g/kg), NaCl (7 g/kg), slow-releasing urea (8 g/kg), and a vitamin–mineral premix (5 g/kg). In the YC diet, yeast culture was included at 10 g/kg DM by replacing an equal amount of corn germ meal to maintain similar nutrient density between treatments. Yeast culture was procured from Xi’an Xinhanbao Biotechnology Co., Ltd., Xi’an, China. Diets were analyzed for DM, OM, NDF, ADF, EE, CP, and starch, and the analyzed nutrient composition is summarized in Table S1. Briefly, on a DM basis, the CON and YC diets contained (g/100 g DM) DM 89.4 and 90.3, OM 93.2 and 92.9, NDF 22.7 and 23.1, ADF 10.1 and 10.9, EE 2.18 and 2.45, CP 15.2 and 15.6, and starch 29.0 and 29.1, respectively. Dietary rumen-degraded protein (RDP) was calculated based on China Feed Database [71] degradability values, averaging 8.98 and 9.26 g/100 g DM for CON and YC, respectively.

Diets were supplied as pelleted TMR. Lambs of the YC treatment experienced a 14‑day transition phase fed a stepwise replacement of the basal diet with the experimental diet, a 7‑day pre‑feeding phase fed the final experimental diet, followed by a 63‑day experiment period. Lambs were housed in individual pens (0.75 m × 1.5 m) and fed their diets twice daily at 08:00 and 17:00, allowing up to 10% refusals, with ad libitum access to water.

### Sample collection and determination

Six lambs per treatment were randomly selected for total fecal collection using bags attached to the lambs from days 48 to 52 of the experiment, with total fecal output daily weighed for each lamb; a composite sample per lamb representing 10% of the daily fecal mass was prepared by pooling feces from the five consecutive days for proximate analysis, while a separate aliquot for CP determination was preserved with 50 mL of 10% (v/v) HCl, and both feed offered and rejected by each lamb were weighed and sampled throughout the fecal collecting period. Standard protocols from AOAC (2000) [72] were followed to analyze DM, ash, and CP. Specifically, DM was assessed by oven-drying samples at 105 ℃ (10 h), while ash content was obtained through incineration in a muffle furnace at 550 °C (6 h). A Kjeldahl analyzer (FOSS Kjeltec™ Distillator, Denmark) was employed to quantify CP [73]. For fiber analysis, NDF and ADF were measured using an ANKOM 200i semi-automatic fiber analyzer (ANKOM Tech, New York, USA), in accordance with the methodology described by Van Soest et al. [74]. Acid detergent lignin (ADL) was determined using the ANKOM filter bag technique following the manufacturer’s protocol [75]. Briefly, after ADF determination, dried residues in filter bags were incubated in 72% (w/w) H₂SO₄ for 3 h with agitation at 30-min intervals, rinsed with water until neutral, rinsed with acetone (3 min), and oven-dried at 105 ℃ for 4 h. Bags were then ashed at 525 ℃ for 3 h, and ADL was calculated with blank bag corrections as specified in the protocol. Hemicellulose was calculated as NDF − ADF, and cellulose as ADF − ADL [76].

On d 63, rumen contents were collected from each lamb via an oral stomach tube before feeding and at 2.5 h and 6 h after the morning feeding. To minimize saliva contamination, the first 50 mL of rumen contents was discarded, and subsequent contents were collected for analysis. Rumen contents were filtered through four layers of gauze, and pH was immediately measured using a portable pH meter (PHB-4; Shanghai Leici, China). Filtered rumen fluid samples were acidified with 25% (w/v) metaphosphoric acid, transferred to centrifuge tubes, and stored at − 20 ℃ for VFA analysis. Concentration of NH_4_^+^–N was determined using a phenol–hypochlorite colorimetric method based on Broderick and Kang [77] with minor modifications. In brief, NH_4_^+^ in rumen fluid reacted with phenol and alkaline sodium hypochlorite, catalyzed by sodium nitroprusside, to form indophenol blue (Berthelot reaction). After color development for 30 min at 37 ℃, absorbance was measured at 550 nm using a microplate reader (BioTek Instruments, Inc., Epoch, USA). Concentrations were calculated from an ammonium chloride standard curve and expressed as mmol/L. Lactate concentration was determined by the colorimetric method of Barker and Summerson [78] with minor modifications. Briefly, lactate was converted to acetaldehyde by heating in concentrated sulfuric acid, and the acetaldehyde subsequently reacted with copper ions and p-hydroxydiphenyl to form a colored complex. After color development, absorbance was measured at 570 nm, and lactate concentration was calculated from an external standard curve prepared from lactic acid standards. For dissolved gases analysis, 35 mL of freshly collected rumen fluid was drawn into a 60-mL gas-tight plastic syringe. A 10-mL N₂ headspace was created by connecting the sample syringe to a second 20-mL syringe prefilled with 10 mL of ultra-pure N₂ and transferring the gas into the 60 mL syringe. The coupled syringes were then shaken horizontally at 200 rpm for 5 min to establish equilibrium between the aqueous and gaseous phases. After equilibration, the headspace was pushed into evacuated 3-mL vacuumed tubes and for analyzing concentrations of H₂ and CH₄ by gas chromatography (Agilent 7890 A, Agilent Inc., Palo Alto, CA). Dissolved concentrations were calculated from the measured headspace values with the respective Ostwald coefficients for H₂ and CH_4_ at 39 ℃ [79]. For microbial DNA analysis, 10 mL aliquots of rumen fluid were snap-frozen in liquid nitrogen and stored at − 80 ℃ until DNA extraction for metagenomic sequencing.

### Metagenomic sequencing and bioinformatics

Microbial DNA was extracted from each rumen fluid sample following the methodology described by Ma et al. [80]. DNA concentration and purity were assessed with a NanoDrop 2000 spectrophotometer (Thermo Fisher Scientific Inc., Waltham, USA) and an Agilent 4200 TapeStation (Agilent Technologies, Inc., Santa Clara, USA). DNA was quantified by Qubit fluorometry (Thermo Fisher Scientific Inc., Waltham, USA) and a commercial kit (Ultima Pro DNA Library Prep; Illumina, Inc., San Diego, USA) was used for library construction following the instructions of the manufacturer. Libraries were sequenced with paired-end 150 bp reads on a DNBSEQ-T7 platform (BGI Genomics Co., Ltd., Beijing, China) by Tsingke Biotechnology Co., Ltd., Beijing, China.

Contigs were binned into metagenome-assembled genomes (MAGs) using MetaBAT2 [81]. An integrative rumen microbiome catalog along with MAGs achieved in the current study was then dereplicated with dRep v3.5 [82] to retain high‑quality genomes (≥ 80% completeness; < 10% contamination) at a 95% average nucleotide identity threshold. Taxonomic annotation was performed on the refined genomes using the gtdbtk v2.4 against GTDB Release 214 [83], completing the workflow from functional profiling to species rank classification.

Microbial genes were identified with Prodigal v2.6.3 [84], and a non‑redundant gene catalog was generated using CD‑HIT v4.8.1 [85]. Functional annotation and genome reconstruction were performed with bioinformatics tools against multiple databases. KO annotation was conducted with KofamScan v1.3.0 against the KEGG database (accessed on 1 June 2024). Genes encoding CAZymes were identified using HMMER v3.2.1 [86] against the CAZy database v11 [87].

Abundances of genes and microbial taxa were quantified with the GEM v3.6.1 [88] and normalized to transcripts per million (TPM) [89], which converts raw read counts to relative expression units per million bases by adjusting for gene length and library size. Genome-resolved functional profiling is constrained by MAG recovery (incompleteness), potential binning/taxonomic assignment errors, and annotation uncertainty for conserved enzyme families; additionally, eukaryotic genomes are not represented in the MAG set, so inferred hydrogenase profiles primarily reflect bacterial contributions.

### Statistics

Normally distributed variables with single sampling time including performance indicators and nutrient digestibility in CON and YC treatments were compared using t-test. Normally distributed variables with multiple sampling times, including pH and VFA concentration, were analyzed with linear mixed‑effects models with treatment, sampling time, and their interaction as fixed effects. Sampling time was also set as a repeated factor with AR (1) covariance structure. For metagenomic data that violated normality assumptions, the DESeq2 method [90] was applied, and adjusted *P*-values were obtained using its default settings [91].

Permutational ANOVA based on the Bray–Curtis dissimilarity matrix [92] was performed using the vegan package [93], with 9999 permutations set. Spearman’s rank correlation was used to assess relationships between microbial profiles and metabolite concentrations. Statistical significance was defined as *P* < 0.05. All statistical analyses were performed in JMP Pro 18 (SAS Institute, Cary, NC, USA), except that the DESeq2 analysis and permutational ANOVA was performed in R v4.2.2 (https://cran.r-project.org).

## Supplementary Information

Supplementary Material 1.

Supplementary Material 2. 

Supplementary Material 3. 

Supplementary Material 4. 

Supplementary Material 5. 

Supplementary Material 6. 

## Data Availability

The metagenomic raw sequences have been deposited at the China National Center for Bioinformation (https://ngdc.cncb.ac.cn/) under the bioProject of PRJCA040155.

## References

[1] Bramley E, Lean IJ, Fulkerson WJ, Stevenson MA, Rabiee AR, Costa ND. The definition of acidosis in dairy herds predominantly fed on pasture and concentrates. J Dairy Sci. 2008;91(1):308–21. 10.3168/jds.2006-601. 18096953 10.3168/jds.2006-601

[2] Plaizier JC, Krause DO, Gozho GN, Mcbride BW. Subacute ruminal acidosis in dairy cows: the physiological causes, incidence and consequences. Vet J. 2008;176(1):21–31. 10.1016/j.tvjl.2007.12.016. 18329918 10.1016/j.tvjl.2007.12.016

[3] Amin AB, Mao S. Influence of yeast on rumen fermentation, growth performance and quality of products in ruminants: a review. Anim Nutr. 2021;7(1):31–41. 10.1016/j.aninu.2020.10.005. 33997329 10.1016/j.aninu.2020.10.005 PMC8110857

[4] Zhong RZ, Fang Y, Zhou DW, Sun XZ, Zhou CS, He YQ. Pelleted total mixed ration improves growth performance of fattening lambs. Anim Feed Sci Technol. 2018;242:127–34. 10.1016/j.anifeedsci.2018.06.008.

[5] Zhang Z, Wang L, Li Q, Li F, Ma Z, Li F, et al. Effects of dietary forage neutral detergent fiber and rumen degradable starch ratios on chewing activity, ruminal fermentation, ruminal microbes and nutrient digestibility of Hu sheep fed a pelleted total mixed ration. J Anim Sci. 2024;102:skae100. 10.1093/jas/skae100. 38581217 10.1093/jas/skae100 PMC11017508

[6] Qi P, Wang L. Effect of adding yeast cultures to high-grain conditions on production performance, rumen fermentation profile, microbial abundance, and immunity in goats. Animals (Basel). 2024;14(12):1799. 10.3390/ani14121799. 38929418 10.3390/ani14121799 PMC11200607

[7] Xiao JX, Alugongo GM, Chung R, Dong SZ, Li SL, Yoon I, et al. Effects of *Saccharomyces cerevisiae* fermentation products on dairy calves: ruminal fermentation, gastrointestinal morphology, and microbial community. J Dairy Sci. 2016;99(7):5401–12. 10.3168/jds.2015-10563. 27157569 10.3168/jds.2015-10563

[8] Wang X, Li F, Zhang N, Ungerfeld E, Guo L, Zhang X, et al. Effects of supplementing a yeast culture in a pelleted total mixed ration on fiber degradation, fermentation parameters, and the bacterial community in the rumen of sheep. Anim Feed Sci Technol. 2023;296:115565. 10.1016/j.anifeedsci.2022.115565.

[9] Zhang S, Geng Y, Ling Y, Wang D, Hu G. Yeast culture is beneficial for improving the rumen fermentation and promoting the growth performance of goats in summer. Fermentation (Basel). 2024;10(6):307. 10.3390/fermentation10060307.

[10] Zhu W, Wei Z, Xu N, Yang F, Yoon I, Chung Y, et al. Effects of *Saccharomyces cerevisiae* fermentation products on performance and rumen fermentation and microbiota in dairy cows fed a diet containing low quality forage. J Anim Sci Biotechnol. 2017;8:36. 10.1186/s40104-017-0167-3. 28465826 10.1186/s40104-017-0167-3 PMC5408399

[11] Liang J, Liu S, Zhang R, Chang J, Lv L, Nabi M, et al. Yeast culture enhances long-term fermentation of corn straw by ruminal microbes for volatile fatty acid production: performance and mechanism. J Environ Manage. 2024;370:122736. 10.1016/j.jenvman.2024.122736. 39362162 10.1016/j.jenvman.2024.122736

[12] Ungerfeld EM. Metabolic hydrogen flows in rumen fermentation: principles and possibilities of interventions. Front Microbiol. 2020;11:589. 10.3389/fmicb.2020.00589. 32351469 10.3389/fmicb.2020.00589 PMC7174568

[13] Mackie RI, Kim H, Kim NK, Cann I. Hydrogen production and hydrogen utilization in the rumen: key to mitigating enteric methane production. Anim Biosci. 2024;37(2):323–36. 10.5713/ab.23.0294. 38186257 10.5713/ab.23.0294 PMC10838669

[14] Ma ZY, Zhang XM, Wang M, Wang R, Jiang ZY, Tan ZL, et al. Molecular hydrogen produced by elemental magnesium inhibits rumen fermentation and enhances methanogenesis in dairy cows. J Dairy Sci. 2019;102(6):5566–76. 10.3168/jds.2018-15647. 30981486 10.3168/jds.2018-15647

[15] Greening C, Geier R, Wang C, Woods LC, Morales SE, Mcdonald MJ, et al. Diverse hydrogen production and consumption pathways influence methane production in ruminants. ISME J. 2019;13(10):2617–32. 10.1038/s41396-019-0464-2. 31243332 10.1038/s41396-019-0464-2 PMC6776011

[16] Li QS, Wang R, Ma ZY, Zhang XM, Jiao J, Zhang ZG, et al. Dietary selection of metabolically distinct microorganisms drives hydrogen metabolism in ruminants. ISME J. 2022;16(11):2535–46. 10.1038/s41396-022-01294-9. 35931768 10.1038/s41396-022-01294-9 PMC9562222

[17] Xu Z, Yang L, Chen H, Liu S, Li X, Li S, et al. *Saccharomyces cerevisiae* and *Kluyveromyces marxianus* yeast co-cultures modulate the ruminal microbiome and metabolite availability to enhance rumen barrier function and growth performance in weaned lambs. Anim Nutr. 2024;19:139–52. 10.1016/j.aninu.2024.06.005. 39635413 10.1016/j.aninu.2024.06.005 PMC11615919

[18] Stewart RD, Auffret MD, Warr A, Walker AW, Roehe R, Watson M. Compendium of 4,941 rumen metagenome-assembled genomes for rumen microbiome biology and enzyme discovery. Nat Biotechnol. 2019;37(8):953–61. 10.1038/s41587-019-0202-3. 31375809 10.1038/s41587-019-0202-3 PMC6785717

[19] Mao J, Wang L. Rumen acidosis in ruminants: a review of the effects of high-concentrate diets and the potential modulatory role of rumen foam. Front Vet Sci. 2025;12:1595615. 10.3389/fvets.2025.1595615. 40496917 10.3389/fvets.2025.1595615 PMC12148896

[20] Li MM, White RR, Guan LL, Harthan L, Hanigan MD. Metatranscriptomic analyses reveal ruminal pH regulates fiber degradation and fermentation by shifting the microbial community and gene expression of carbohydrate-active enzymes. Anim Microbiome. 2021;3(1):32. 10.1186/s42523-021-00092-6. 33892824 10.1186/s42523-021-00092-6 PMC8063335

[21] Russell JB. Effect of extracellular pH on growth and proton motive force of *Bacteroides succinogenes*, a cellulolytic ruminal bacterium. Appl Environ Microbiol. 1987;53(10):2379–83. 10.1128/aem.53.10.2379-2383.1987. 2827568 10.1128/aem.53.10.2379-2383.1987 PMC204116

[22] Russell JB. Resistance of *Streptococcus bovis* to acetic acid at low pH: relationship between intracellular pH and anion accumulation. Appl Environ Microbiol. 1991;57(1):255–9. 10.1128/aem.57.1.255-259.1991. 2036013 10.1128/aem.57.1.255-259.1991 PMC182694

[23] Dias ALG, Freitas JA, Micai B, Azevedo RA, Greco LF, Santos JEP. Effect of supplemental yeast culture and dietary starch content on rumen fermentation and digestion in dairy cows. J Dairy Sci. 2018;101(1):201–21. 10.3168/jds.2017-13241. 29103715 10.3168/jds.2017-13241

[24] Knollinger SE, Poczynek M, Miller B, Mueller I, de Almeida R, Murphy MR, et al. Effects of autolyzed yeast supplementation in a high-starch diet on rumen health, apparent digestibility, and production variables of lactating Holstein cows. Animals (Basel). 2022;12(18):2445. 10.3390/ani12182445. 36139305 10.3390/ani12182445 PMC9495083

[25] de Assis Lage CF, Räisänen SE, Melgar A, Nedelkov K, Chen X, Oh J, et al. Comparison of two sampling techniques for evaluating ruminal fermentation and microbiota in the planktonic phase of rumen digesta in dairy cows. Front Microbiol. 2020;11:618032. 10.3389/fmicb.2020.618032. 33424820 10.3389/fmicb.2020.618032 PMC7785721

[26] Newbold CJ, Wallace RJ, Mcintosh FM. Mode of action of the yeast *Saccharomyces cerevisiaeas* a feed additive for ruminants. Br J Nutr. 1996;76(2):249–61. 10.1079/BJN19960029. 8813899 10.1079/bjn19960029

[27] Firkins JL, Mitchell KE. Invited review: rumen modifiers in today’s dairy rations. J Dairy Sci. 2023;106(5):3053–71. 10.3168/jds.2022-22644. 36935236 10.3168/jds.2022-22644

[28] Martin SA. Manipulation of ruminal fermentation with organic acids: a review. J Anim Sci. 1998;76(12):3123–32. 10.2527/1998.76123123x. 9928618 10.2527/1998.76123123x

[29] Firkins JL, Henderson EL, Duan H, Pope PB. International Symposium on Ruminant Physiology: current perspective on rumen microbial ecology to improve fiber digestibility. J Dairy Sci. 2025;108(7):7511–29. 10.3168/jds.2024-25863. * *Presented as part of the Gastrointestinal Microbial Ecology, the Microbiome, and Gut Physiology Spanning from Microbial-Host Interactions to an Update on Methane Production and Mineral Interactions session at the 2024 International Symposium on Ruminant Physiology, August 2024. 39701529 10.3168/jds.2024-25863

[30] Su M, Wang H, Shi H, Li Q, Zhang Y, Li T, et al. Yeast products mediated ruminal subenvironmental microbiota, and abnormal metabolites and digestive enzymes regulated rumen fermentation function in sheep. Animals (Basel). 2022;12(22):3221. 10.3390/ani12223221. 36428448 10.3390/ani12223221 PMC9686794

[31] Li Z, Hu Y, Li H, Lin Y, Cheng M, Zhu F, et al. Effects of yeast culture supplementation on milk yield, rumen fermentation, metabolism, and bacterial composition in dairy goats. Front Vet Sci. 2024;11:1447238. 10.3389/fvets.2024.1447238. 39170629 10.3389/fvets.2024.1447238 PMC11336828

[32] Carro MD, Ungerfeld EM. Utilization of Organic Acids to Manipulate Ruminal Fermentation and Improve Ruminant Productivity. In: Puniya AK, Singh R, Kamra DN, editors. Rumen Microbiology: From Evolution to Revolution. New Delhi: Springer India; 2015. p. 177–97.

[33] Ungerfeld EM, Kohn RA, Wallace RJ, Newbold CJ. A meta-analysis of fumarate effects on methane production in ruminal batch cultures. J Anim Sci. 2007;85(10):2556–63. 10.2527/jas.2006-674. 17565060 10.2527/jas.2006-674

[34] Ungerfeld EM, Forster RJ. A meta-analysis of malate effects on methanogenesis in ruminal batch cultures. Anim Feed Sci Technol. 2011;166–167:282–90. 10.1016/j.anifeedsci.2011.04.018.

[35] Yi S, Tian X, Qin X, Zhang Y, Guan S, Chen Z, et al. Effects of yeast cultures on growth performance, fiber digestibility, ruminal dissolved gases, antioxidant capacity and immune activity of beef cattle. Animals (Basel). 2025. 10.3390/ani15101452. 40427329 10.3390/ani15101452 PMC12108184

[36] Chaucheyras-Durand F, Walker ND, Bach A. Effects of active dry yeasts on the rumen microbial ecosystem: past, present and future. Anim Feed Sci Technol. 2008;145(1):5–26. 10.1016/j.anifeedsci.2007.04.019.

[37] Cai L, Hartanto R, Xu Q, Zhang J, Qi D. *Saccharomyces cerevisiae* and *Clostridium butyricum* could improve B-Vitamin production in the rumen and growth performance of heat-stressed goats. Metabolites. 2022;12(8):766. 10.3390/metabo12080766. 36005638 10.3390/metabo12080766 PMC9414707

[38] Nagaraja TG, Newbold CJ, van Nevel CJ, Demeyer DI. Manipulation of ruminal fermentation. In: Hobson PN, Stewart CS, editors. The Rumen Microbial Ecosystem. Dordrecht: Springer, Netherlands; 1997. p. 523–632.

[39] Russell JB, Wallace RJ. Energy-yielding and energy-consuming reactions. In: Hobson PN, Stewart CS, editors. The Rumen Microbial Ecosystem. Dordrecht: Springer, Netherlands; 1997. p. 246–82.

[40] Russell JB. Rumen microbiology and its role in ruminant nutrition: Department of Microbiology, Cornell University; 2002.

[41] Averianova LA, Balabanova LA, Son OM, Podvolotskaya AB, Tekutyeva LA. Production of Vitamin B2 (Riboflavin) by microorganisms: an overview. Front Bioeng Biotechnol. 2020. 10.3389/fbioe.2020.570828. 33304888 10.3389/fbioe.2020.570828 PMC7693651

[42] Chowdhury NP, Mowafy AM, Demmer JK, Upadhyay V, Koelzer S, Jayamani E, et al. Studies on the mechanism of electron bifurcation catalyzed by electron transferring flavoprotein (Etf) and butyryl-CoA dehydrogenase (Bcd) of *Acidaminococcus fermentans*. J Biol Chem. 2014;289(8):5145–57. 10.1074/jbc.M113.521013. 24379410 10.1074/jbc.M113.521013 PMC3931072

[43] Newbold CJ, de la Fuente G, Belanche A, Ramos-Morales E, Mcewan NR. The Role of Ciliate Protozoa in the Rumen. Front Microbiol. 2015;6. 10.3389/fmicb.2015.01313. 10.3389/fmicb.2015.01313 PMC4659874 26635774

[44] Seshadri R, Leahy SC, Attwood GT, Teh KH, Lambie SC, Cookson AL, et al. Cultivation and sequencing of rumen microbiome members from the Hungate1000 Collection. Nat Biotechnol. 2018;36(4):359–67. 10.1038/nbt.4110. 29553575 10.1038/nbt.4110 PMC6118326

[45] Kelly WJ, Mackie RI, Attwood GT, Janssen PH, Mcallister TA, Leahy SC. Hydrogen and formate production and utilisation in the rumen and the human colon. Anim Microbiome. 2022;4(1):22. 10.1186/s42523-022-00174-z. 35287765 10.1186/s42523-022-00174-z PMC8919644

[46] David LA, Maurice CF, Carmody RN, Gootenberg DB, Button JE, Wolfe BE, et al. Diet rapidly and reproducibly alters the human gut microbiome. Nature. 2014;505(7484):559–63. 10.1038/nature12820. 24336217 10.1038/nature12820 PMC3957428

[47] Malik PK, Trivedi S, Kolte AP, Mohapatra A, Biswas S, Bhattar A, et al. Comparative rumen metagenome and CAZyme profiles in cattle and buffaloes: Implications for methane yield and rumen fermentation on a common diet. Microorganisms. 2023. 10.3390/microorganisms12010047. 38257874 10.3390/microorganisms12010047 PMC10818812

[48] Firrincieli A, Minuti A, Cappelletti M, Ferilli M, Ajmone-Marsan P, Bani P, et al. Structural and functional analysis of the active cow rumen’s microbial community provides a catalogue of genes and microbes participating in the deconstruction of cardoon biomass. Biotechnol Biofuels Bioprod. 2024;17(1):53. 10.1186/s13068-024-02495-4. 38589938 10.1186/s13068-024-02495-4 PMC11003169

[49] Wang J, Zhao G, Zhuang Y, Chai J, Zhang N. Yeast (*Saccharomyces cerevisiae*) culture promotes the performance of fattening sheep by enhancing nutrients digestibility and rumen development. Fermentation (Basel). 2022;8(12):719. 10.3390/fermentation8120719.

[50] Auer E, Lazuka A, Huguenin-Bizot B, Jehmlich N, Déjean S, Lombard V, et al. Horizontal metaproteomics and CAZymes analysis of lignocellulolytic microbial consortia selectively enriched from cow rumen and termite gut. ISME Commun. 2023. 10.1038/s43705-023-00339-0.

[51] Terry SA, Badhan A, Wang Y, Chaves AV, Mcallister TA. Fibre digestion by rumen microbiota — a review of recent metagenomic and metatranscriptomic studies. Can J Anim Sci. 2019;99(4):678–92. 10.1139/cjas-2019-0024.

[52] Weimer PJ. Degradation of cellulose and hemicellulose by ruminal microorganisms. Microorganisms. 2022;10(12):2345. 10.3390/microorganisms10122345. 36557598 10.3390/microorganisms10122345 PMC9785684

[53] Kelly WJ, Henderson G, Pacheco DM, Li D, Reilly K, Naylor GE, et al. The complete genome sequence of *Eubacterium limosum* SA11, a metabolically versatile rumen acetogen. Stand Genomic Sci. 2016;11:26. 10.1186/s40793-016-0147-9. 26981167 10.1186/s40793-016-0147-9 PMC4791908

[54] Mewis K, Lenfant N, Lombard V, Henrissat B. Dividing the large Glycoside Hydrolase Family 43 into subfamilies: a motivation for detailed enzyme characterization. Appl Environ Microbiol. 2016;82(6):1686–92. 10.1128/AEM.03453-15. 26729713 10.1128/AEM.03453-15 PMC4784025

[55] Dai X, Tian Y, Li J, Su X, Wang X, Zhao S, et al. Metatranscriptomic analyses of plant cell wall polysaccharide degradation by microorganisms in the cow rumen. Appl Environ Microbiol. 2015;81(4):1375–86. 10.1128/AEM.03682-14. 25501482 10.1128/AEM.03682-14 PMC4309707

[56] Li Z, Wang X, Zhang Y, Yu Z, Zhang T, Dai X, et al. Genomic insights into the phylogeny and biomass-degrading enzymes of rumen ciliates. ISME J. 2022;16(12):2775–87. 10.1038/s41396-022-01306-8. 35986094 10.1038/s41396-022-01306-8 PMC9666518

[57] Andersen TO, Altshuler I, Vera-Ponce DLA, Walter JM, Mcgovern E, Keogh K, et al. Metabolic influence of core ciliates within the rumen microbiome. ISME J. 2023;17(7):1128–40. 10.1038/s41396-023-01407-y 10.1038/s41396-023-01407-y PMC10284877 37169869

[58] Zhu X, Liu B, Xiao J, Guo M, Zhao S, Hu M, et al. Effects of different roughage diets on fattening performance, meat quality, fatty acid composition, and rumen microbe in steers. Front Nutr. 2022;9:885069. 10.3389/fnut.2022.885069. 35799586 10.3389/fnut.2022.885069 PMC9253607

[59] Satter LD, Slyter LL. Effect of ammonia concentration on rumen microbial protein production in vitro. Br J Nutr. 1974;32(2):199–208. 10.1079/BJN19740073. 4472574 10.1079/bjn19740073

[60] Schwab CG, Broderick GA. A 100-year review: protein and amino acid nutrition in dairy cows. J Dairy Sci. 2017;100(12):10094–112. 10.3168/jds.2017-13320. 29153157 10.3168/jds.2017-13320

[61] Ramos TR, Souza KAD, Prado RM, Ornaghi MG, Stuani OF, Casetta J, et al. Ruminal metabolism, blood parameters and animal behavior of bulls submitted to sub-acute ruminal acidosis (SARA) receiving different buffers in high-concentrate diets. Acta Sci Anim Sci. 2025;48(1):e75262. 10.4025/actascianimsci.v48i1.75262.

[62] Arik HD, Gulsen N, Hayirli A, Alatas MS. Efficacy of*Megasphaera elsdenii* inoculation in subacute ruminal acidosis in cattle. J Anim Physiol Anim Nutr (Berl). 2019;103(2):416–26. 10.1111/jpn.13034. 30588673 10.1111/jpn.13034

[63] Pathak N, Guan H, Fan P, Sultana H, Arriola K, Oyebade A, et al. Comparing rumen fluid collection methods on fermentation profile and microbial population in lactating dairy cows. JDS Commun. 2025;6(1):34–8. 10.3168/jdsc.2024-0566. 39877175 10.3168/jdsc.2024-0566 PMC11770309

[64] Janssen PH. Influence of hydrogen on rumen methane formation and fermentation balances through microbial growth kinetics and fermentation thermodynamics. Anim Feed Sci Technol. 2010;160(1–2):1–22. 10.1016/j.anifeedsci.2010.07.002.

[65] Russell JB. The importance of pH in the regulation of ruminal acetate to propionate ratio and methane production in vitro. J Dairy Sci. 1998;81(12):3222–30. 10.3168/jds.S0022-0302(98)75886-2. 9891267 10.3168/jds.S0022-0302(98)75886-2

[66] Lechartier C, Peyraud JL. The effects of starch and rapidly degradable dry matter from concentrate on ruminal digestion in dairy cows fed corn silage-based diets with fixed forage proportion. J Dairy Sci. 2011;94(5):2440–54. 10.3168/jds.2010-3285. 21524536 10.3168/jds.2010-3285

[67] Klevenhusen F, Zebeli Q. A review on the potentials of using feeds rich in water‐soluble carbohydrates to enhance rumen health and sustainability of dairy cattle production. J Sci Food Agric. 2021;101(14):5737–46. 10.1002/jsfa.11358. 34091911 10.1002/jsfa.11358

[68] Genthner BR, Davis CL, Bryant MP. Features of rumen and sewage sludge strains of *Eubacterium limosum*, a methanol-and H_2_-CO_2_-utilizing species. Appl Environ Microbiol. 1981;42(1):12–9. 10.1128/aem.42.1.12-19.1981. 6791591 10.1128/aem.42.1.12-19.1981 PMC243953

[69] Chaucheyras F, Fonty G, Bertin G, Gouet P. In vitro H2 utilization by a ruminal acetogenic bacterium cultivated alone or in association with an archaea methanogen is stimulated by a probiotic strain of *Saccharomyces cerevisiae*. Appl Environ Microbiol. 1995;61(9):3466–7. 10.1128/aem.61.9.3466-3467.1995. 7574654 10.1128/aem.61.9.3466-3467.1995 PMC167624

[70] Leclerc M, Elfoul-Bensaid L, Bernalier A. Effect of yeast extract on growth and metabolism of H2-utilizing acetogenic bacteria from the human colon. Curr Microbiol. 1998;37(3):166–71. 10.1007/s002849900358. 9688815 10.1007/s002849900358

[71] China Feed Database. https://www.chinafeeddata.org.cn/admin/Login/index.html. Accessed 15 Jan 2026.

[72] AOAC H W. International a: official methods of analysis of the AOAC international. Arlington County,VA,USA: The Association; 2000.

[73] Wang L, Sun X, Liang J, Ma Z, Li F, Hao S, et al. Machine learning model interpretability using SHAP values: applied to the task of classifying and predicting the nutritional content of different cuts of mutton. Food Chemistry: X. 2025;29:102739. 10.1016/j.fochx.2025.102739. 40686892 10.1016/j.fochx.2025.102739 PMC12275142

[74] Van Soest PJ, Robertson JB, Lewis BA. Methods for dietary fiber, neutral detergent fiber, and nonstarch polysaccharides in relation to animal nutrition. J Dairy Sci. 1991;74(10):3583–97. 10.3168/jds.S0022-0302(91)78551-2. 1660498 10.3168/jds.S0022-0302(91)78551-2

[75] Ankom T. Method 8: Determining acid detergent lignin in beakers. In: ANKOM Technology Method 08/05, Ankom Technology Macedon, NY; 2005

[76] Stypinski JD, Weiss WP, Carroll AL, Kononoff PJ. Effect of acid detergent lignin concentration for diets formulated to be similar in neutral detergent fiber content on energy utilization in lactating Jersey cows. J Dairy Sci. 2024;107(8):5699–708. 10.3168/jds.2023-24318. 38608940 10.3168/jds.2023-24318

[77] Broderick GA, Kang JH. Automated simultaneous determination of ammonia and total amino acids in ruminal fluid and in vitro media. J Dairy Sci. 1980;63(1):64–75. 10.3168/jds.S0022-0302(80)82888-8. 7372898 10.3168/jds.S0022-0302(80)82888-8

[78] Barker SB, Summerson WH. The colorimetric determination of lactic acid in biological material. J Biol Chem. 1941;138(2):535–54. 10.1016/S0021-9258(18)51379-X.

[79] Wang M, Sun XZ, Janssen PH, Tang SX, Tan ZL. Responses of methane production and fermentation pathways to the increased dissolved hydrogen concentration generated by eight substrates in in vitro ruminal cultures. Anim Feed Sci Technol. 2014;194:1–11. 10.1016/j.anifeedsci.2014.04.012.

[80] Ma ZY, Zhang XM, Wang R, Wang M, Liu T, Tan ZL. Effects of chemical and mechanical lysis on microbial DNA yield, integrity, and downstream amplicon sequencing of rumen bacteria and protozoa. Front Microbiol. 2020;11:581227. 10.3389/fmicb.2020.581227. 33304329 10.3389/fmicb.2020.581227 PMC7701101

[81] Kang DD, Li F, Kirton E, Thomas A, Egan R, An H, et al. MetaBAT 2: an adaptive binning algorithm for robust and efficient genome reconstruction from metagenome assemblies. PeerJ. 2019;7:e7359. 10.7717/peerj.7359. 31388474 10.7717/peerj.7359 PMC6662567

[82] Olm MR, Brown CT, Brooks B, Banfield JF. dRep: a tool for fast and accurate genomic comparisons that enables improved genome recovery from metagenomes through de-replication. ISME J. 2017;11(12):2864–8. 10.1038/ismej.2017.126. 28742071 10.1038/ismej.2017.126 PMC5702732

[83] Chaumeil P, Mussig AJ, Hugenholtz P, Parks DH. GTDB-Tk: a toolkit to classify genomes with the Genome Taxonomy Database. Bioinformatics. 2020;36(6):1925–7. 10.1093/bioinformatics/btz848. 10.1093/bioinformatics/btz848 PMC7703759 31730192

[84] Hyatt D, Chen GL, Locascio PF, Land ML, Larimer FW, Hauser LJ. Prodigal: prokaryotic gene recognition and translation initiation site identification. BMC Bioinformatics. 2010;11:119. 10.1186/1471-2105-11-119. 20211023 10.1186/1471-2105-11-119 PMC2848648

[85] Li W, Godzik A. Cd-hit: a fast program for clustering and comparing large sets of protein or nucleotide sequences. Bioinformatics. 2006;22(13):1658–9. 10.1093/bioinformatics/btl158. 16731699 10.1093/bioinformatics/btl158

[86] Yu DS, Lee D, Kim SK, Lee CH, Song JY, Kong EB. Algorithm for predicting functionally equivalent proteins from BLAST and HMMER searches. J Microbiol Biotechnol. 2012;22(8):1054. 10.4014/jmb.1203.03050. 22713980 10.4014/jmb.1203.03050

[87] Nguyen STC, Freund HL, Kasanjian J, Berlemont R. Function, distribution, and annotation of characterized cellulases, xylanases, and chitinases from CAZy. Appl Microbiol Biotechnol. 2018;102(4):1629–37. 10.1007/s00253-018-8778-y. 29359269 10.1007/s00253-018-8778-y PMC5806127

[88] Ashish N, Dewan P, Ambite J, Toga AW. GEM: The GAAIN Entity Mapper. In: Ashish N, Ambite J, editors. Cham: Springer International Publishing; 2015; 13–27 10.1007/978-3-319-21843-4_2 PMC4671774 26665184

[89] Mortazavi A, Williams BA, Mccue K, Schaeffer L, Wold B. Mapping and quantifying mammalian transcriptomes by RNA-Seq. Nat Methods. 2008;5(7):621–8. 10.1038/nmeth.1226. 18516045 10.1038/nmeth.1226 PMC13303166

[90] Love MI, Huber W, Anders S. Moderated estimation of fold change and dispersion for RNA-seq data with DESeq2. Genome Biol. 2014. 10.1186/s13059-014-0550-8. 25516281 10.1186/s13059-014-0550-8 PMC4302049

[91] Benjamini Y, Hochberg Y. Controlling the false discovery rate: A practical and powerful approach to multiple testing. J R Stat Soc B Stat Methodol. 1995;57(1):289–300. 10.1111/j.2517-6161.1995.tb02031.x.

[92] Anderson MJ. A new method for non-parametric multivariate analysis of variance. Austral Ecol. 2001;26(1):32–46. 10.1111/j.1442-9993.2001.01070.pp.x.

[93] Oksanen J, Simpson GL, F B, R K, P L, P M, et al. vegan: Community Ecology Package. R package version 2.7–0. 2025. https://vegandevs.github.io/vegan/. Accessed 20 Mar 2025.

