# Supplementary material for: Yeast culture promotes butyrate produced fibrolytic bacteria as intracellular hydrogen sink in the rumen
Source: Microbiome. 2026 May 25;14:183. doi: 10.1186/s40168-026-02436-3 (PMC13383528; doi:10.1186/s40168-026-02436-3)
Supplement: Supplementary file 2 — Supplementary Material 1. [file 40168_2026_2436_MOESM1_ESM.docx]

**Supplementary information**

**Table captions:**

Table S1. Ingredients and chemical compositions of experimental diets.

**Figure captions:**

Figure S1 Effects of yeast culture supplementation on prokaryotes diversity (A, alpha diversity; B, beta diversity) and community composition at the species level (C) in rumen of growing lambs (n = 10). Alpha and beta diversities were evaluated at strain level. The Principal Coordinates Analysis (PCoA) was constructed based on a Bray-Curtis dissimilarity matrix to visualize beta diversity patterns across samples.

Figure S2. Effects of yeast culture supplementation on prokaryotes alpha diversity, beta diversity at the phylum, class, order, family and genus level in rumen of growing lambs.

Figure S3. Acetate production genes distributions assigned genus level.

Figure S4. Propionante production genes distributions assigned genus level.

Figure S5. Butyrate production genes distributions assigned genus level.

Figure S6. Phylogenomic of prokaryotes MAGs enriched by CON and YC treatment.

Table S1. Ingredients and chemical compositions of experimental diets.

| Item | CON | YC |
| --- | --- | --- |
| Ingredient composition, g/kg DM | | |
| Corn straw | 200 | 200 |
| Corn grain | 250 | 250 |
| Barley | 250 | 250 |
| Corn germ meal | 105 | 95 |
| Soybean meal | 80 | 80 |
| Cottonseed meal | 50 | 50 |
| Yeast culture^1^ | 0 | 10 |
| Limestone | 15 | 15 |
| Molasses | 30 | 30 |
| NaCl | 7 | 7 |
| Slow-releasing urea^2^ | 8 | 8 |
| Premix^3^ | 5 | 5 |
| Total | 1000 | 1000 |
| Nutrient composition^4^, g/100g DM | | |
| DM | 89.4 | 90.3 |
| NDF | 22.7 | 23.1 |
| ADF | 10.1 | 10.9 |
| ADL | 4.10 | 4.31 |
| OM | 93.2 | 92.9 |
| EE | 2.18 | 2.45 |
| CP | 15.2 | 15.6 |
| RDP^5^ | 8.98 | 9.26 |
| Starch | 29.0 | 29.1 |
| ME, MJ/kg | 10.0 | 11.9 |

^1^ Yeast culture was procured from Xi’an Xinhanbao Biotechnology Co., Ltd., Xi’an, China, and included 12% moisture, 0.8% mannan, 16% crude protein, 12% ash, 1.28% citrate, 0.44% malate, and 0.27% succinate according to the manufacturer.

^2^ Slow-releasing urea was procured form Wuwei Hengda Animal Husbandry Co., Ltd., Wuwei, China, and included 32% N and 12% moisture according to the manufacturer.

^3^ The premix was provided following as per kg of feed DM: Fe 25 mg, Mn 40 mg, Zn 40 mg, Cu 8 mg, I 0.3 mg, Se 0.2 mg, Co 0.1 mg, vitamin A 940 IU, vitamin D 111 IU, vitamin E 20 IU.

^4^ DM, dry matter; OM, organic matter; CP, crude protein; NDF, neutral detergent fibre; ADF, acid detergent fibre; EE, ether extract; ME, metabolizable energy.

^5^ Dietary RDP content was calculated from the ingredient composition according to the Tables of Feed Composition and Nutritive Values in China (http://www.chinafeeddata.org.cn/; accessed 28 December 2025).





Figure S1 Effects of yeast culture supplementation on prokaryotes diversity (A, alpha diversity; B, beta diversity) and community composition at the species level (C) in rumen of growing lambs (n = 10). Alpha and beta diversities were evaluated at strain level. The Principal Coordinates Analysis (PCoA) was constructed based on a Bray-Curtis dissimilarity matrix to visualize beta diversity patterns across samples.





Figure S2. Effects of yeast culture supplementation on prokaryotes alpha diversity, beta diversity at the phylum, class, order, family and genus level in rumen of growing lambs (*n* = 10). Alpha and beta diversities were evaluated at strain level. The Principal Coordinates Analysis (PCoA) was constructed based on a Bray-Curtis dissimilarity matrix to visualize beta diversity patterns across samples.


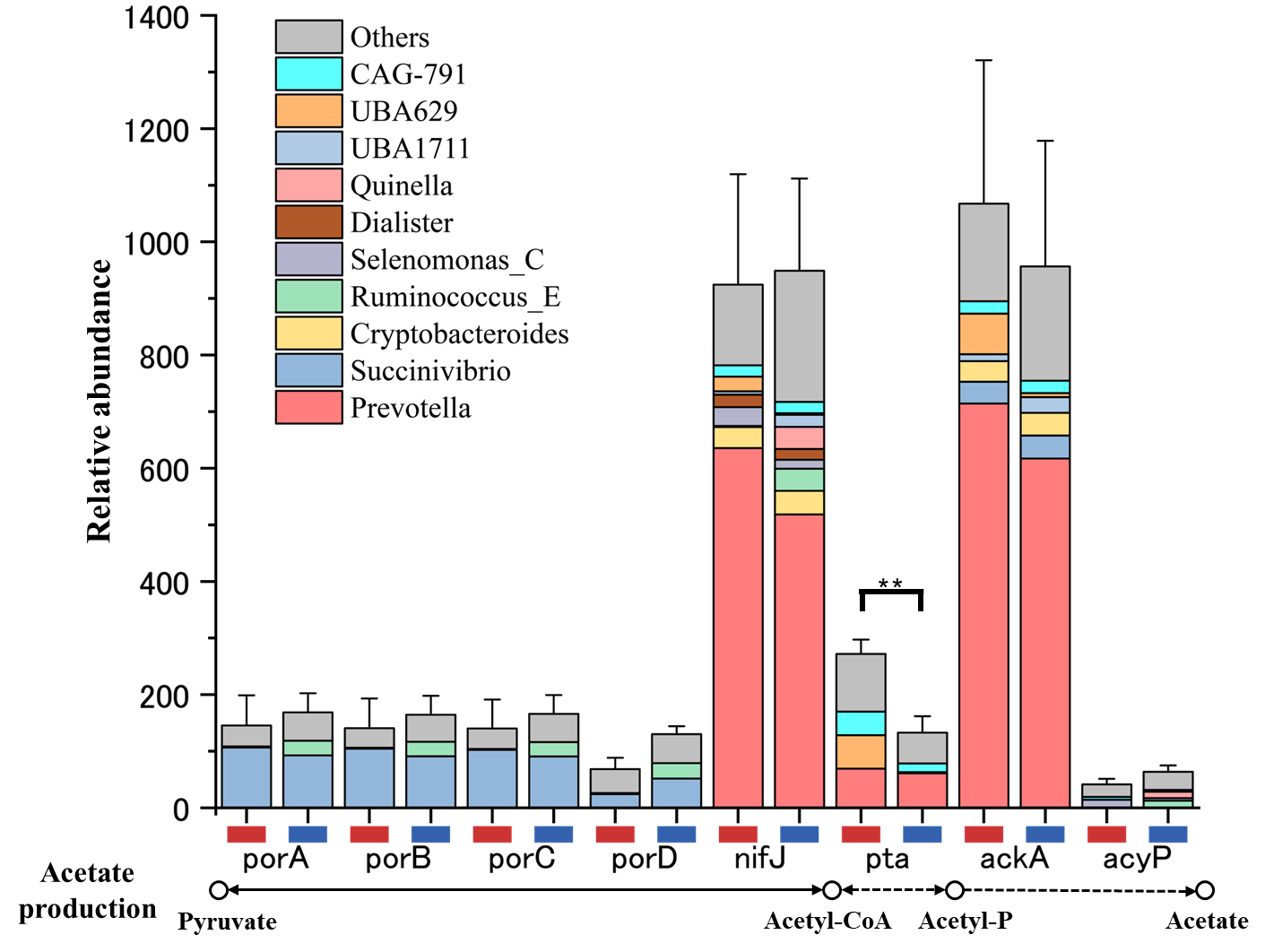


Figure S3. Acetate production genes distributions assigned genus level. **p* < 0.05, ***p* < 0.01, ****p* < 0.001, *n* = 10/group.


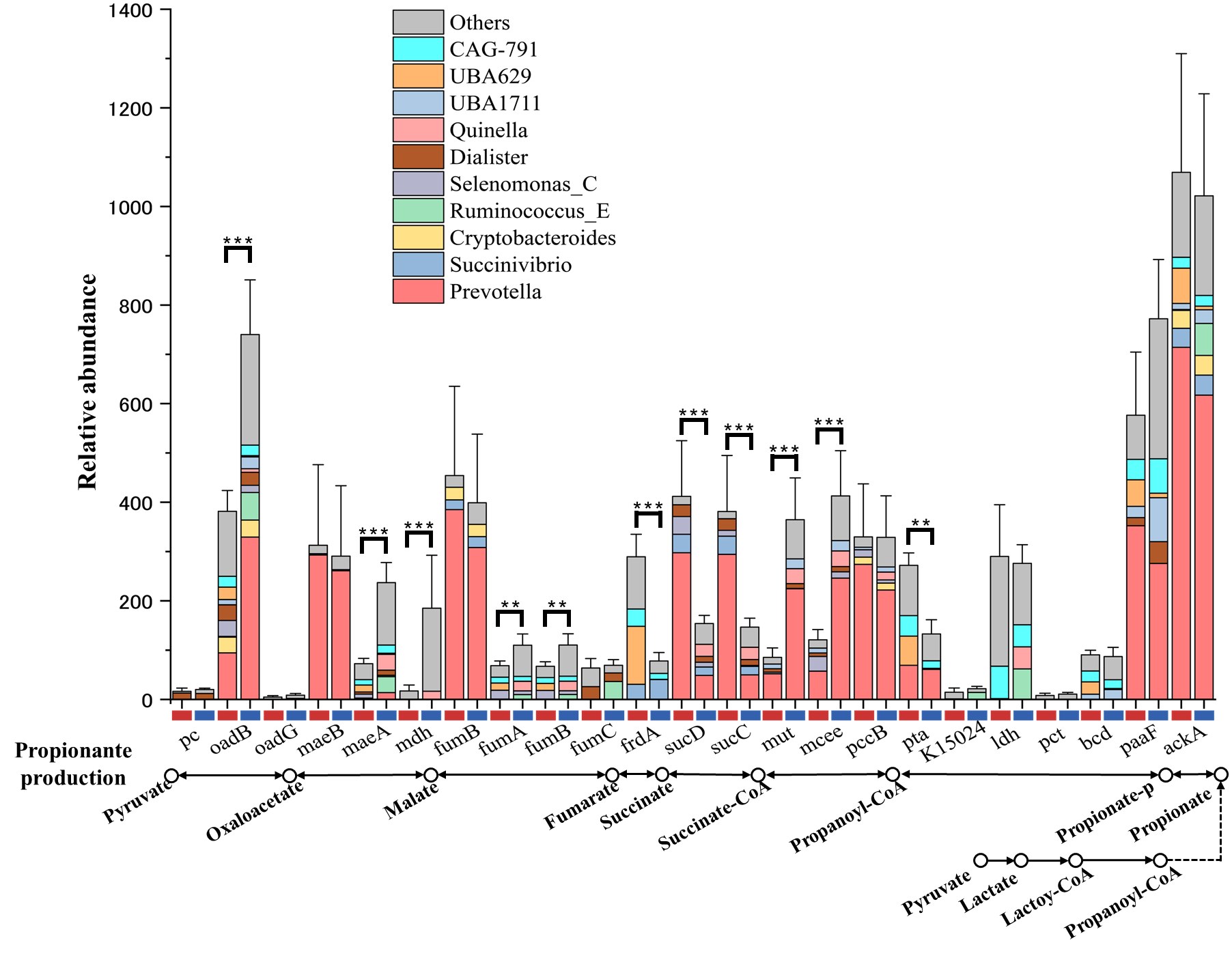


Figure S4. Propionante production genes distributions assigned genus level. **p* < 0.05, ***p* < 0.01, ****p* < 0.001, *n* = 10/group.


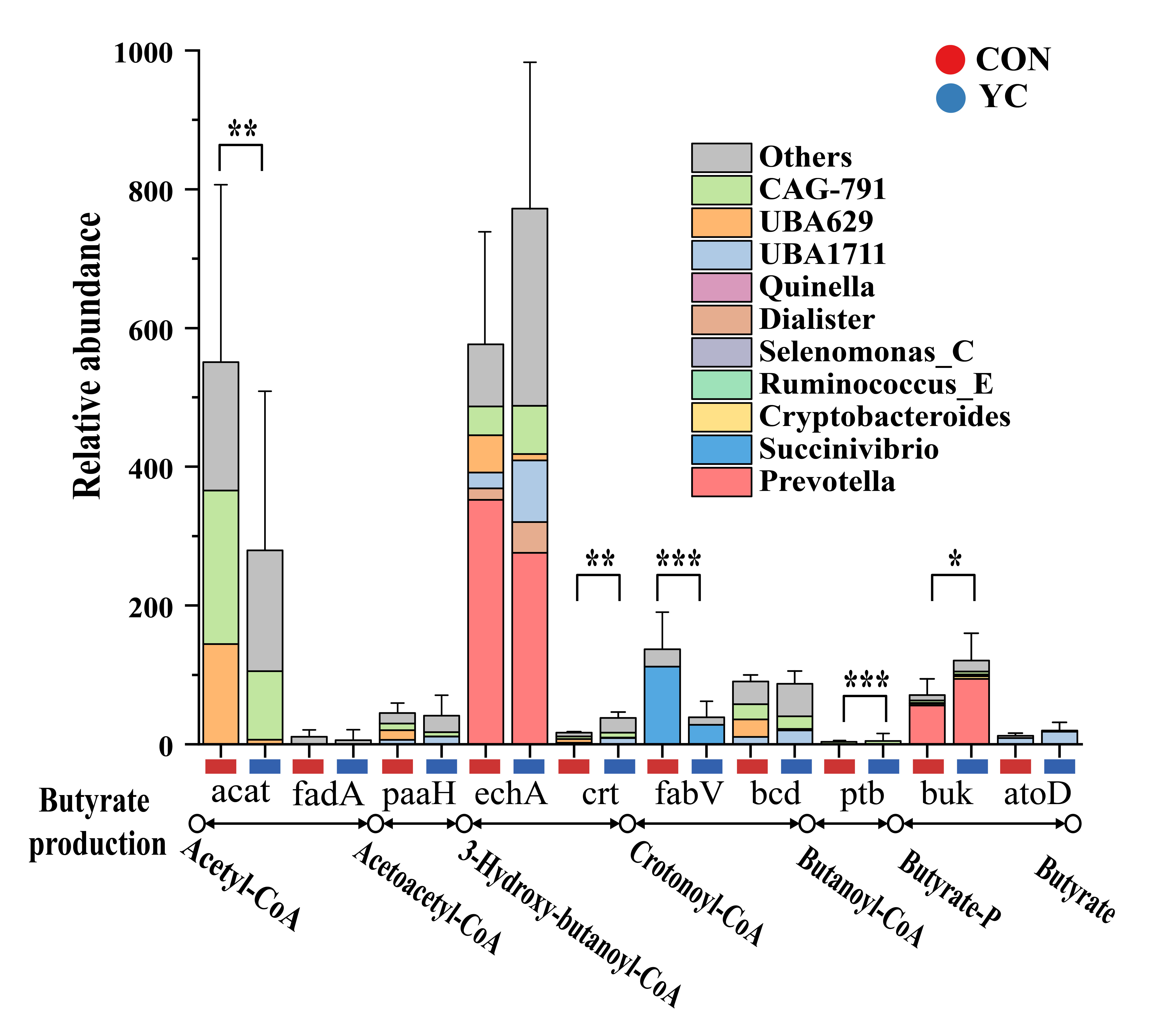


Figure S5. Butyrate production genes distributions assigned genus level. **p* < 0.05, ***p* < 0.01, ****p* < 0.001, *n* = 10/group.





Figure S6. Phylogenomic of prokaryotes MAGs enriched by CON and YC treatment. Phylogenetic tree of 2,750 prokaryotes annotated for the following genes: encoding carbohydrate-active enzymes (CAZymes), hydrogenases, and enzymes involved in butyrogenesis. Fermentative hydrogenases (group A1, A2 and B FeFe-hydrogenases), electron-bifurcating hydrogenases (group A3 FeFe-hydrogenases), energy-converting hydrogenases (bidirectional; group 4f NiFe-hydrogenases), respiratory hydrogenases (group 1d NiFe-hydrogenases), sensory hydrogenases (group C FeFe-hydrogenases).
